# A GmNRF5a–GmCERK1–GmCAK1 module mediates chitin/chitosan‐triggered immune response in soybean

**DOI:** 10.1111/jipb.70042

**Published:** 2025-10-06

**Authors:** Guangzheng Sun, Jun Chen, Tang Li, Qinsheng Zhu, Xinrui Li, Xuan Mi, Wenxia Wang, Zhichao Zhang, Keyi Huang, Ruoting Yao, Bo Yang, Wenwu Ye, Kaixuan Duan, Zhenchuan Ma, Ke Yu, Yiming Wang, Suomeng Dong, Yan Wang, Heng Yin, Yuanchao Wang

**Affiliations:** ^1^ Department of Plant Pathology Nanjing Agricultural University Nanjing 210095 China; ^2^ Key Laboratory of Soybean Disease and Pest Control (Ministry of Agriculture and Rural Affairs) Nanjing Agricultural University Nanjing 210095 China; ^3^ Suzhou Academy of Agricultural Sciences Suzhou 234000 China; ^4^ Dalian Engineering Research Center for Carbohydrate Agricultural Preparations, Dalian Technology Innovation Center for Green Agriculture, Liaoning Provincial Key Laboratory of Carbohydrates, Dalian Institute of Chemical Physics Chinese Academy of Sciences Dalian 116023 China; ^5^ State Key Laboratory of Crop Stress Adaptation and Improvement, Henan Joint International Laboratory for Crop Multi‐Omics Research, School of Life Sciences Henan University Kaifeng 475004 China

**Keywords:** CERK1‐associated kinase 1 (CAK1), CHITIN ELICITOR RECEPTOR KINASE1 (CERK1), chitin oligosaccharide (CTOS), chitosan oligosaccharide (CSOS), NOD factor receptor protein 5a (NFR5a), soybean

## Abstract

Chitin and its deacetylated derivative chitosan are the major components of fungal cell walls and are recognized by plant pattern‐recognition receptors (PRRs) as pathogen‐associated molecular patterns that induce innate immunity. Recognition of chitin oligosaccharide (CTOS) in Arabidopsis (*Arabidopsis thaliana*) and rice (*Oryza sativa*) requires the membrane‐localized lysin‐motif (LysM)‐domain‐containing receptors AtLYK5 and OsCEBiP, respectively. However, the mechanism underlying chitosan oligosaccharide (CSOS)‐induced plant immunity remains unclear. In this study, we determined that CTOS and CSOS trigger immune responses and boost disease resistance in soybean (*Glycine max*) through the LysM‐domain‐containing protein GmNRF5a and its co‐receptor GmCERK1. Surprisingly, both GmNFR5a and GmCERK1 bind directly to CTOS and CSOS, with distinct binding sites. The receptor‐like kinase GmCAK1 acts downstream of GmCERK1 and is essential for CTOS/CSOS‐mediated immune activation. Overall, these findings uncovered how soybean plants respond to CSOS and initiate immune signaling, demonstrating that soybean exploits shared immune sectors to transduce immune signals triggered by CTOS/CSOS, paving the way for the development of disease‐resistant crops with broad‐spectrum resistance.

## Graphical abstract

In soybeans, the GmNFR5a‐GmCERK1‐GmCAK1 module regulates immune responses triggered by chitin oligosaccharides and chitosan oligosaccharides from fungal pathogens. This reveals a shared upstream signaling mechanism for distinct microbial patterns and provides valuable genetic targets for improving broad‐spectrum disease resistance in crops.

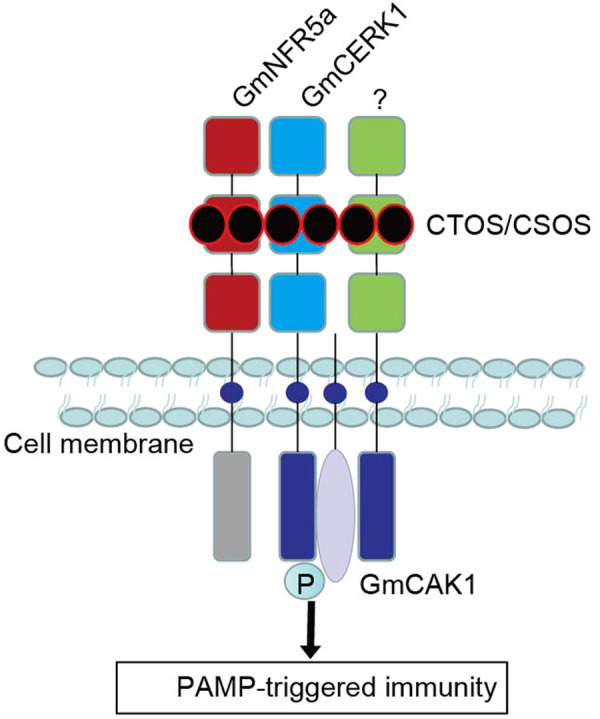

## INTRODUCTION

Plants in natural environments are continually exposed to potential pathogens such as bacteria, fungi, oomycetes, and viruses. To prevent pathogen invasion, plants have evolved multi‐layered immune systems, including pathogen‐associated molecular pattern (PAMP)‐triggered immunity (PTI) and effector‐triggered immunity (ETI) (Chisholm et al., 2006). Pathogen‐associated molecular pattern‐triggered immunity is generally initiated via recognition of conserved potential PAMPs, such as bacterial flagellin and peptidoglycan (PGN), fungal chitin, and oomycete XEG1 and INF1 (Jones and Dangl, 2006; Silipo et al., 2010; Sun et al., 2022; Chen et al., 2023), by pattern‐recognition receptors (PRRs) on the plant cell surface (Segonzac and Zipfel, 2011). A well studied subset of the large and expanding repertoire of PRRs is a group of proteins containing extracellular leucine‐rich repeat (LRR) or lysin‐motif (LysM) domains. Pathogen‐associated molecular pattern recognition triggers rapid and transient activation of a series of early immune responses, including a reactive oxygen species (ROS) burst, mitogen‐activated protein kinase (MAPK) phosphorylation, calcium influx, callose deposition, and expression of pathogenesis‐related genes (Macho and Zipfel, 2014; Xiao et al., 2024).

Chitin, a linear polysaccharide composed of *β*‐1,4‐linked *N*‐acetylglucosamine residues, is a major component of the fungal cell wall. It can be cross‐linked with other polysaccharides to protect the fungal cell wall from hydrolysis and maintain the structural integrity of hyphae (Kombrink et al., 2011; Goldman and Vicencio, 2012). During a fungal invasion, plants secrete chitinases that degrade the chitin layers, producing short‐chain chitin oligosaccharides (CTOS); these are recognized as a PAMP by transmembrane LysM receptor‐like kinases or receptor‐like proteins, thereby initiating PTI (Liu et al., 2012; Akamatsu et al., 2013; Hayafune et al., 2014). To dampen chitin‐induced plant immune responses, many fungi secrete chitin de‐*N*‐acetylases that convert chitin to chitosan, which is less susceptible to chitinase digestion, thus evading cell wall degradation (El Gueddari et al., 2002; Cord‐Landwehr et al., 2016; Gao et al., 2019). Interestingly, chitosan oligosaccharides (CSOS), derived from chitosan hydrolysis by chemical degradation, are potent plant immune elicitors (Aziz et al., 2006; Povero et al., 2011; He et al., 2018). Chitin oligosaccharide and CSOS, collectively known as chitooligosaccharides, can be prepared in large amounts by enzymolysis of chitin or chitosan and are used in agriculture to trigger disease resistance in crops (Sun et al., 2018; Huang et al., 2021). Given their pivotal role in safeguarding plants, the utilization of chitooligosaccharides in agriculture, particularly for plant disease management, is garnering vital interest.

In Arabidopsis (*Arabidopsis thaliana*), the extracellular domains (ECD) of the single‐pass transmembrane receptor kinases CHITIN ELICITOR RECEPTOR KINASE 1 (CERK1) and LysM RECEPTOR KINASE 5 (LYK5) contain three LysM domains (Liu et al., 2012). The second LysM domain (LysM2) of AtLYK5 and AtCERK1 directly binds to CTOS, with AtLYK5 having a higher binding affinity than AtCERK1 (Cao et al., 2014). Binding of chitin to AtCERK1 triggers auto‐phosphorylation of the receptor at threonine and tyrosine residues (Petutschnig et al., 2010), initiating a cascade of plant defense responses (Miya et al., 2007; Wan et al., 2008; Liu et al., 2012). The rice (*Oryza sativa*) chitin receptor CHITIN ELICITOR BINDING PROTEIN (OsCEBiP) is a receptor‐like protein (RLP) with a LysM‐containing extracellular domain and a Cys‐rich domain (CRD) at the N‐terminal, coupled with a short transmembrane helix at the C‐terminal (Kaku et al., 2006; Liu et al., 2016). OsCEBiP has high‐affinity for CTOS (Kaku et al., 2006; Hayafune et al., 2014) and forms a heterooligomeric receptor complex with the CRD kinase OsCERK1, which is pivotal for chitin recognition (Shimizu et al., 2010). OsCERK1 directly binds CTOS and is indispensable for CTOS‐induced signaling (Xu et al., 2023), though with significantly lower affinity than OsCEBiP (Liu et al., 2016). Notably, CERK1 is a central receptor in *Medicago truncatula* and *Lotus japonicus* that bridges chitin recognition to immune defense and symbiotic signaling (Bozsoki et al., 2017; Feng et al., 2019). Its dual functionality and partnership with other LysM‐RLKs (e.g., LYR4, LYK8) enable precise discrimination between pathogenic and beneficial fungi, ensuring appropriate physiological responses (Zhang et al., 2024; Simonsen et al., 2025). In addition, the kinase activity of CERK1 is essential for the chitin signaling pathway (Wang et al., 2017). Chitin recognition is promptly translated into cellular immune responses through intracellular signaling mechanisms mainly driven by dynamic protein–protein interactions and a series of phosphorylation events. In Arabidopsis, several receptor‐like cytoplasmic kinases (RLCKs), such as PBS‐like 19 (PBL19), PBL27, and BOTRYTIS INDUCED KINASE 1 (BIK1), can assemble into complexes with AtLYK5‐AtCERK1, thereby facilitating the transduction of chitin‐induced signals (Gong et al., 2020). A similar scenario unfolds in rice, in which OsRLCK185 and OsRLCK176 interact with and are phosphorylated by autophosphorylated OsCERK1 in a chitin‐responsive manner (Yamaguchi et al., 2013; Ao et al., 2014; Shinya et al., 2014). However, despite these findings in the model systems of Arabidopsis and rice, the mechanism underlying CTOS‐induced and CSOS‐induced plant immunity in soybean (*Glycine max*) remains unclear.

Phytopathogenic oomycetes are responsible for many destructive diseases of important crops worldwide. For instance, soybean root and stem rot caused by *Phytophthora sojae* leads to millions of dollars of losses annually (Tyler, 2007). Previous research indicated that chitin is a component of the zoospore and sporangia cell wall in *P. sojae* (Cheng et al., 2019), but the mechanisms by which soybean plants respond to chitin have yet to be fully elucidated. Here we show that the exogenous application of chitooligosaccharides (CTOS/CSOS) leads to a marked enhancement of resistance against *P. sojae* in soybean. Furthermore, this response depended on the LysM‐domain‐containing membrane proteins GmNFR5a and GmCERK1. The immune responses initiated by CTOS/CSOS were significantly attenuated in soybean plants in which *GmNFR5a* or *GmCERK1* had been silenced. We established that the ectodomains of GmNFR5a (GmNFR5a^ECD^) and GmCERK1 (GmCERK1^ECD^) directly bind to chitooligosaccharides. The critical binding sites of GmNFR5a and CSOS, as well as GmCERK1 and CSOS were identified; however, these sites are not essential for CTOS binding. Moreover, we found that the soybean receptor‐like kinase GmCAK1 is critical for propagating the chitooligosaccharide‐triggered signaling cascade and that GmCAK1 modulates this process through the auto‐phosphorylation of GmCERK1. Taken together, our findings shed light on how soybean plants sense fungal cell wall components, paving the way for the development of disease‐resistant crops with broad‐spectrum disease resistance.

## RESULTS

### Chitooligosaccharides trigger immune responses and enhance *P. sojae* resistance in soybean

**Figure 1:**
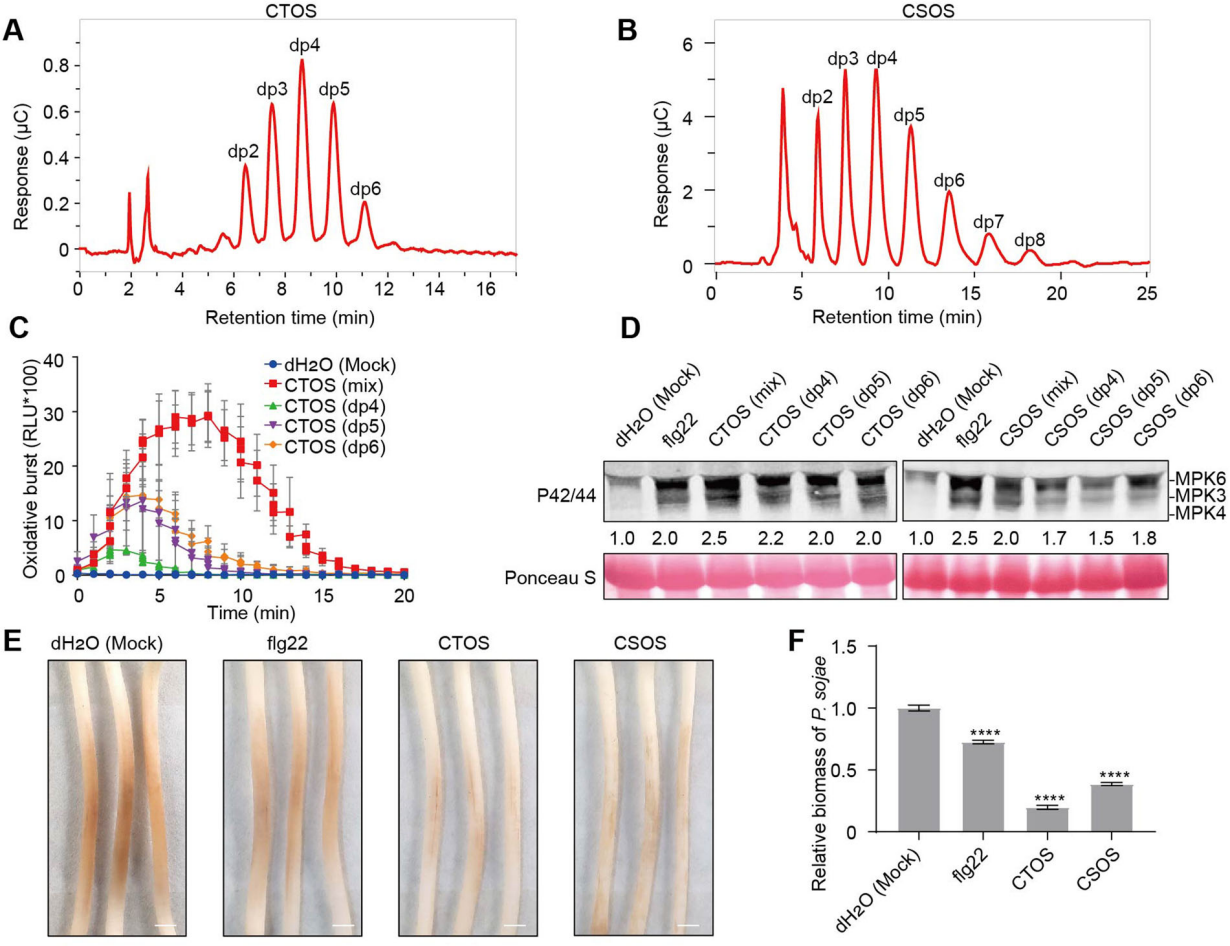
**Chitooligosaccharides trigger soybean immune responses and induce plant resistance against** ***Phytophthora sojae*** **(A**, **B)** ESI‐MS and HILIC‐HPLC analyses reveal differences in the polymerization of chitin oligosaccharide (CTOS) **(A)** or chitosan oligosaccharide (CSOS) **(B)**. **(C)** Reactive oxygen species (ROS) production triggered by CTOS (50 mg/L) in leaf discs of soybean Williams plants, dH_2_O as negative control. Mean relative luminescence unit (RLU) (± *SD*) is shown (*n* = 6). **(D)** Differences in the polymerization of chitooligosaccharides‐triggered mitogen‐activated protein kinase (MAPK) phosphorylation, which was examined in treated soybean leaf discs at 5 min. Total protein was analyzed by immunoblot with an antibody for phosphorylated MPK6/3/4 (P42/44). The total band intensities were quantified using ImageJ software. **(E)** Visual symptoms 48 h after inoculation with *P. sojae* P6497 on soybean etiolated hypocotyls, which were pretreated separately with CTOS/CSOS (dp6, 50 mg/L). Representative photographs are shown. Scale bar: 0.5 cm. **(F)** Relative biomass of *P. sojae* infecting etiolated soybean hypocotyls (at 48 h post inoculation (hpi), as measured by genomic DNA quantitative polymerase chain reaction (qPCR), and normalized to P6497. Asterisks indicate statistically significant differences to control based on Student's *t‐*test (****P ≤ 0.0001). All experiments were repeated three times with similar results.

To investigate the role of CTOS and CSOS in activating plant immunity in soybean, chitooligosaccharides with different degrees of polymerization (dps) were generated by enzymatically digesting shrimp and crab exoskeletons. Chitin oligosaccharide with dps of 2–6 and CSOS with dps of 2–8 were analyzed using ESI‐MS, and then separated and purified by HILIC‐HPLC (Figures 1A, B, S1). Nuclear magnetic resonance (NMR) results suggested that the CSOS were fully deacetylated (Figure S2). Reactive oxygen species burst, detected using a luminol‐based ROS assay, served as an indicator of immune response activation in soybean. Chitin oligosaccharide of dps 2–6 (mix) induced a strong ROS burst. Chitin oligosaccharide (dp5) and CTOS (dp6) elicited a stronger ROS burst than that of CTOS (dp4) (Figure 1C). However, we did not observe a ROS burst following the application of CSOS (dp4–6) in soybean (Figure S3). These results align with previous reports indicating that deacetylation diminishes CTOS‐triggered immune responses in plants, particularly the ROS burst (Gubaeva et al., 2018; Gao et al., 2019). In addition, we observed phosphorylation of MAPKs after treatment with CTOS or CSOS, with flg22 serving as a positive control. As depicted in Figure 1D, CTOS (dp4–6) and CSOS (dp4–6) elicited MAPK phosphorylation levels that were notably higher than those elicited by the control (dH_2_O). Next, we explored whether the application of CTOS and CSOS could enhance soybean resistance against *P. sojae*. Soybean etiolated hypocotyls were treated with flg22, CTOS (dp6, 50 mg/L), or CSOS (dp6, 50 mg/L) 24 h before being inoculated with *P. sojae* zoospores (Figure 1E). Compared with mock‐treated samples (dH_2_O), the biomass of *P. sojae* was significantly lower in hypocotyls treated with CTOS or CSOS (Figure 1F). Taken together, these results show that chitooligosaccharides trigger immune responses and enhance *P. sojae* resistance in soybean.

### GmNRF5a and GmCERK1 are required for CTOS/CSOS‐triggered immune responses in soybean

Previous research has suggested that, in plants such as Arabidopsis and rice, chitin is recognized by LysM domain‐containing proteins (Shimizu et al., 2010; Liu et al., 2012). We therefore conducted an evolutionary analysis of potential LysM‐domain‐containing receptors in angiosperms (Figure S5). We selected two dicotyledonous species, Arabidopsis and soybean, and two monocotyledonous species, rice and maize (*Zea mays*), for phylogenetic analysis. We generated phylogenies based on the amino acid sequences of 84 putative membrane‐localized LysM proteins available from the NCBI database (https://www.ncbi.nlm.nih.gov/Structure/cdd): 47 LysM‐receptor‐like kinases (RLKs) (23 GmLYKs, 5 AtLYKs, 10 OsLYKs, and 9 ZmLYKs) and 37 LysM‐RLPs (14 GmLYPs, 6 AtLYPs, 7 OsLYPs, and 10 ZmLYPs) (Figure S6). All the LYK proteins contained a signal peptide, one to three extracellular LysM domains, a transmembrane domain, and a kinase domain. The LYP proteins consisted of a signal peptide, one to three extracellular LysM domains, and a C‐tail, but lacked a transmembrane domain or kinase domain. These proteins were divided into five groups based on the phylogenetic analysis: OutGroup (OG) and Groups 1–4 (G1–G4) (Figure S6A). The G1 cluster contained the CERK1 homologs from various species. AtCERK1 (AT3G21630), OsCERK1 (Os09g33630), and GmCERK1 (Glyma.20G054500) possess three complete LysM domains in their extracellular regions. However, other CERK1 homologs in soybean, specifically Glyma.15G111300, Glyma.02G270700, and Glyma.14G046200, lack these three complete LysM domains in the extracellular space. The well known CTOS receptors AtLYK5 and OsCEBiP fell into G2 and G4, respectively. GmNFR5a (Glyma.11G063100) belongs to the G2 cluster, but its homologous protein (Glyma.02G000400) lacks three complete LysM domains in the extracellular space. A previous study showed that the extracellular LysM2 domain of OsCEBiP or AtLYK5 is required for chitin binding. Therefore, we conducted an additional phylogenetic analysis based on the amino acid sequences of the LysM2 domain. Again, the LYKs and LYPs were classified into distinct functional groups (Figure S6B). GmNFR5a and AtLYK5 fell into G2, whereas CERK1 and CEBiP were placed in G1 and G4, respectively.

**Figure 2:**
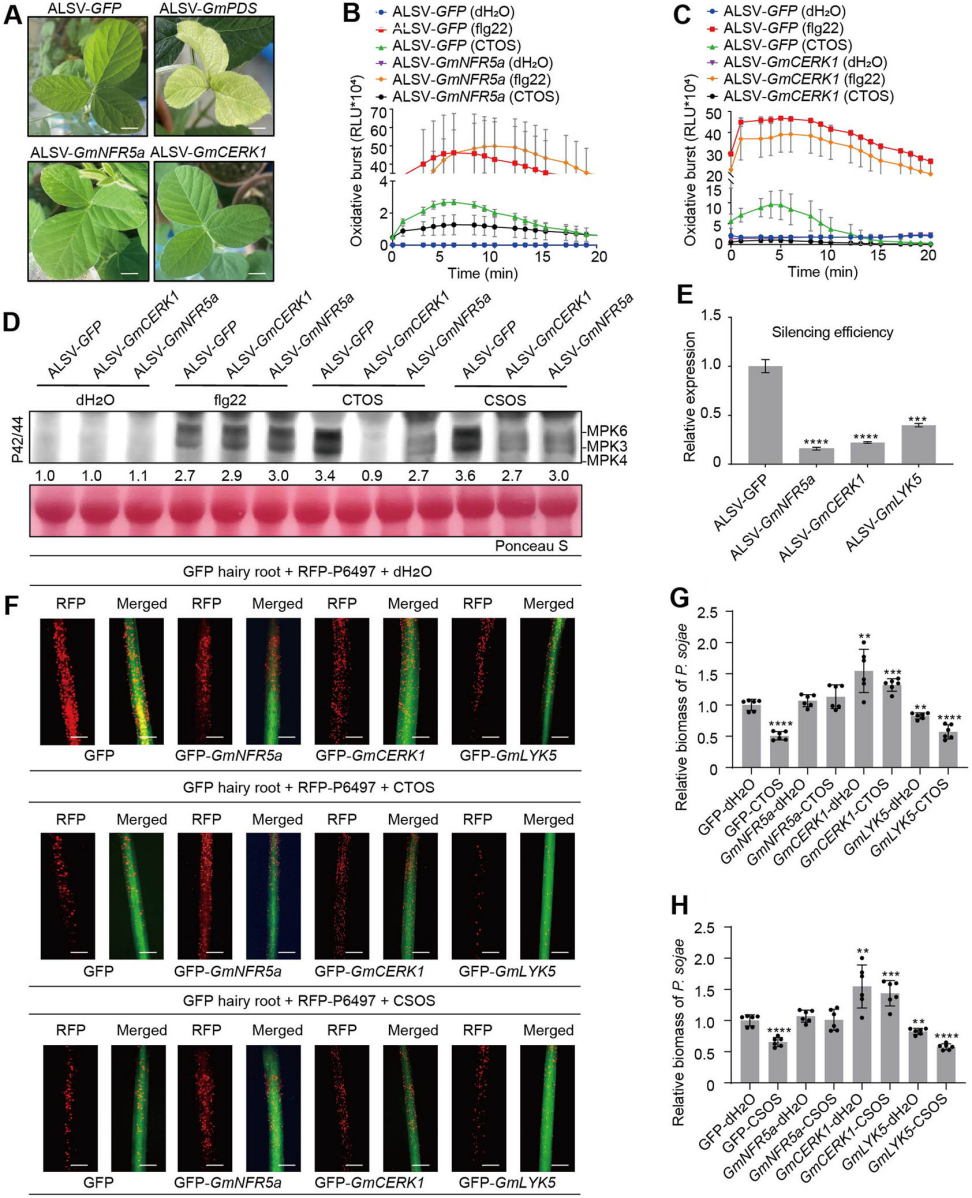
GmNFR5a and GmCERK1 were essential for CTOS/CSOS‐triggered immune responses and disease resistance in soybean **(A)** Plant growth phenotype after gene silencing in soybean plants. After 20 d of inoculation of virus particles on soybean leaves, silenced‐*GmPDS* soybean plants showed albinism. Scale bar: 1.5 cm. **(B**, **C)** Reactive oxygen species (ROS) production triggered by CTOS (50 mg/L) in leaf discs of soybean Zhonghuang 13 plants silenced *GmNFR5a* **(B)** or *GmCERK1* **(C)** gene, flg22 as positive control. Mean relative luminescence unit (RLU) (± *SD*) are shown (*n* = 6). **(D)** CTOS/CSOS‐triggered mitogen‐activated protein kinase (MAPK) phosphorylation in silenced‐Gm*NFR5a* or silenced‐*GmCERK1* transgenic soybean leaf discs at 5 min, flg22 as a positive control. Total protein was analyzed by immunoblot with an antibody for phosphorylated MPK6/3/4 (P42/44). The total band intensities were quantified using ImageJ software. **(E)** Silencing efficiency was quantified by real‐time quantitative polymerase chain reaction (qRT‐PCR) measurement, normalized with *GmCYP2*, and expressed as mean fold changes relative to apple latent spherical virus (ALSV)‐*GFP* treated leaves, which was set as 1. **(F)** Expression levels of Gm*NFR5a* and Gm*CERK1* in soybean hairy roots affect CTOS/CSOS‐triggered defense against *Phytophthora sojae*. Transgenic hairy roots expressing green fluorescent protein (GFP) control, or *GmNFR5a‐*, *GmCERK1‐*, or *GmLYK5‐* specific RNAi construct were inoculated with *P. sojae* zoospores expressing red fluorescent protein (RFP). Oospore production at 48 h post inoculation (hpi) is shown. Six independent experiments gave similar results. Left, RFP; right, merge (RFP + GFP). From top to bottom, three lines of root hairs are treated with dH_2_O, CTOS, and CSOS, respectively. Scale bars, 0.2 mm. **(G**, **H)** The relative biomass of *P. sojae* in infected hairy roots treated by CTOS **(G)** or CSOS **(H)**, measured by genomic DNA qPCR and normalized to the GFP control. In **(E**, **G**, **H)**, asterisks indicate statistically significant differences to control based on Student's *t‐*test (**P ≤ 0.01, ***P ≤ 0.001, ****P ≤ 0.0001). All experiments were repeated three times with similar results.

To clarify CTOS/CSOS‐triggered immune signaling in soybean, we standardized the degree of polymerization for CTOS and CSOS, and selected CTOS (dp6) and CSOS (dp6) according to the abundance in the digested products for further investigation. To determine whether LysM proteins are integral to CTOS/CSOS‐triggered immune responses in soybean, we used the apple latent spherical virus (ALSV) silencing vector to suppress the expression of all genes encoding LysM proteins in soybean (Li et al., 2000; Dong et al., 2022) and assessed the subsequent impact on CTOS‐ and CSOS‐triggered immune responses (Figures 2, S6). The efficacy of ALSV as a gene silencing tool was verified by classic yellowing symptoms in *N. benthamiana* plants inoculated with ALSV‐*CH42* (Chlorata42) (Burch‐Smith et al., 2006; Dong et al., 2022) (Figure S6A). An ALSV‐*GmPDS* construct, which causes leaf photo‐bleaching in soybean (Figure 2A), served as a positive control for experimental validation. Chitin oligosaccharide (dp6)‐triggered ROS burst and MAPK activity developed in the ALSV‐*GFP*‐treated control plants (Figure 2B–D). In contrast, CTOS (dp6)‐induced ROS burst and MAPK activation were obviously compromised or abolished in the *GmCERK1*‐ or *GmNFR5a*‐silenced soybeans (Figure 2B–D). However, silencing these genes had no effect on the ROS burst and MAPK activation triggered by flg22 (Figure 2B–D). Furthermore, MAPK activation in response to CSOS (dp6) was obviously lower in *GmCERK1*‐silenced or *GmNFR5a*‐silenced soybeans than in the control (ALSV‐*GFP*) (Figure 2D). These results suggested that both GmNFR5a and GmCERK1 play an essential role in mediating CTOS/CSOS‐triggered immune responses in soybean.

Utilizing the soybean hairy‐root RNA interference (RNAi) system, we examined the contributions of GmNFR5a and GmCERK1 to root‐based immune responses. Silencing these genes attenuated the CTOS‐induced and CSOS‐induced resistance response to the pathogen *P. sojae* in soybean roots (Figure 2F). When treated with CTOS or CSOS prior to inoculation with *P. sojae*, *GmCERK1*‐silenced plants had a significantly higher biomass of the pathogen than the control plants (green fluorescent protein (GFP)‐dH_2_O). However, *GmNFR5a*‐silenced plants did not exhibit a similar increase in pathogen biomass (Figure 2G, H). Furthermore, silencing *GmLYK5*, an ortholog of the Arabidopsis gene *AtLYK5*, did not markedly impact the CTOS‐ or CSOS‐elicited resistance responses (Figure 2F–H). Nonetheless, slightly less *P. sojae* biomass was observed in *GmLYK5*‐silenced plants pretreated with dH_2_O than in control plants (GFP‐dH_2_O). This finding suggested a possible divergence in function between GmLYK5 and AtLYK5, attributable to their low sequence similarity (Figure S7), resulting in different roles in disease resistance. Collectively, our findings underscore the important roles of both GmNFR5a and GmCERK1 in mediating CTOS/CSOS‐triggered soybean defenses against *P. sojae*.

### Chitin oligosaccharide and CSOS directly bind to GmNFR5a and GmCERK1

**Figure 3:**
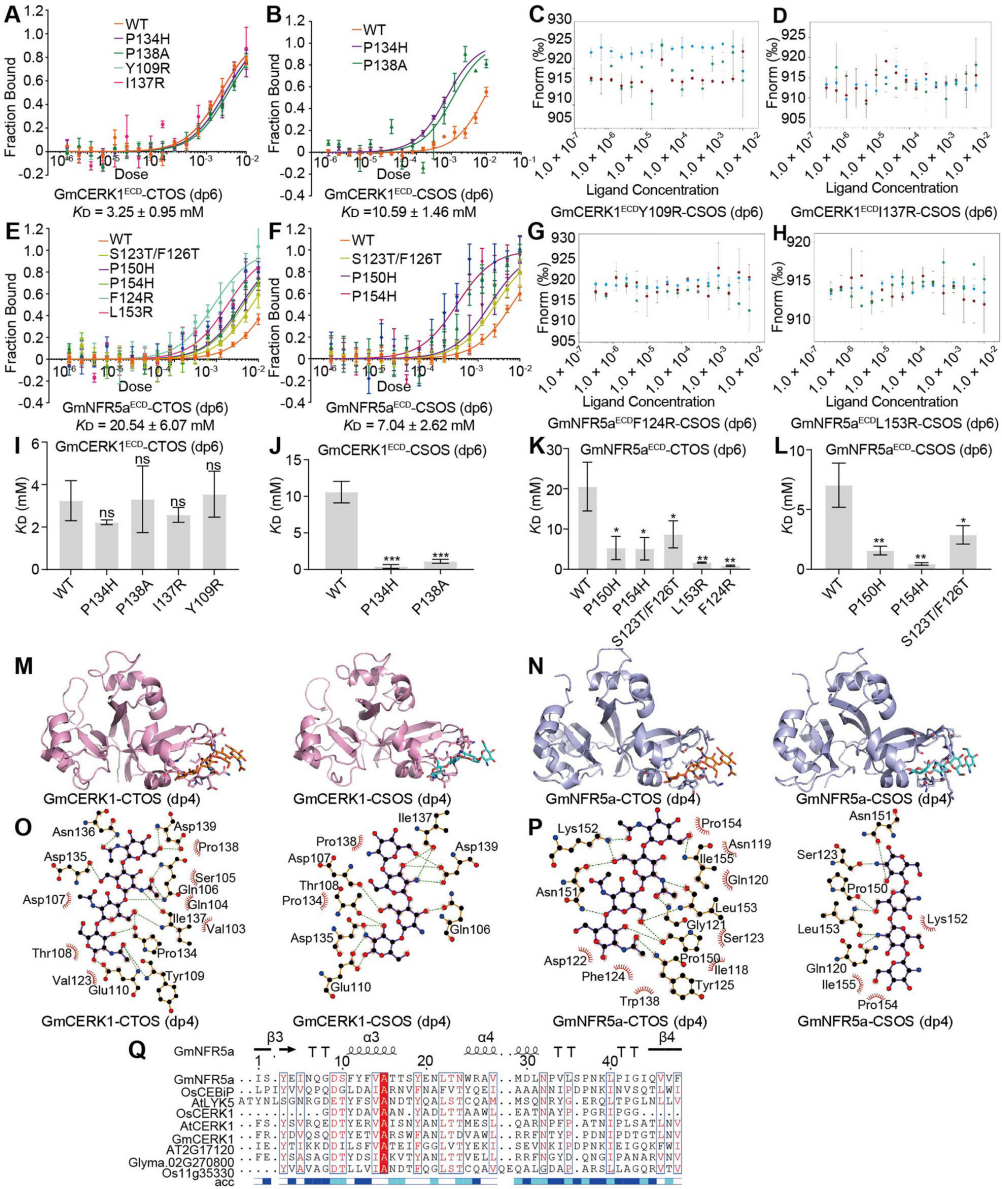
CTOS/CSOS directly binds to GmNFR5a and GmCERK1 **(A)** GmCERK1^ECD^ and GmCERK1^ECD^ mutants bind to CTOS (dp6) *in vitro*. His‐tagged GmCERK1^ECD^ (22.8 kDa) or mutants were incubated with CTOS (dp6). The binding affinity of His‐GmCERK1^ECD^ or mutants with CTOS (dp6) was examined by microscale thermophoresis (MST). Y109R, P134H, I137R, and P138A are different GmCERK1^ECD^ mutant proteins. These proteins were purified from *Pichia pastoris*. **(B)** GmCERK1^ECD^ and GmCERK1^ECD^ mutants bind to CSOS (dp6) *in vitro*. His‐tagged GmCERK1^ECD^ or mutants were incubated with CSOS (dp6). The binding affinity of His‐GmCERK1^ECD^ or mutants with CSOS (dp6) was examined by MST. **(C)** GmCERK1^ECD^Y109R cannot interact with CSOS. His‐tagged GmCERK1^ECD^Y109R was incubated with CSOS (dp6). The binding affinity of His‐GmCERK1^ECD^Y109R with CSOS (dp6) was examined by MST. **(D)** GmCERK1^ECD^I137R cannot interact with CSOS. His‐tagged GmCERK1^ECD^I137R was incubated with CSOS (dp6). The binding affinity of His‐GmCERK1^ECD^Y109R with CSOS (dp6) was examined by MST. **(E)** GmNFR5a^ECD^ and GmNFR5a^ECD^ mutants bind to CTOS (dp6) *in vitro*. His‐tagged GmNFR5a^ECD^ (24.2 kDa) or mutants were incubated with CTOS (dp6). Co‐precipitation of His‐GmNFR5a^ECD^ or mutants with CTOS (dp6) was examined by MST. S123T/F126T, F124R, P150H, L153R, and P154H are different GmNFR5a^ECD^ mutant proteins. **(F)** GmNFR5a^ECD^ and GmNFR5a^ECD^ mutants bind to CSOS (dp6) *in vitro*. His‐tagged GmNFR5a^ECD^ or mutants were incubated with CSOS (dp6). The binding affinity of His‐GmNFR5a^ECD^ or mutants with CSOS (dp6) was examined by MST. **(G)** GmNFR5a^ECD^F124R cannot interact with CSOS. His‐tagged GmNFR5a^ECD^F124R was incubated with CSOS (dp6). The binding affinity of His‐NFR5a^ECD^F124R with CSOS (dp6) was examined by MST. **(H)** GmNFR5a^ECD^L153R cannot interact with CSOS. His‐tagged GmNFR5a^ECD^L153R was incubated with CSOS (dp6). The binding affinity of His‐NFR5a^ECD^L153R with CSOS (dp6) was examined by MST. **(I)** The dissociation constants (*K*_D_) of GmCERK1^ECD^ mutants with CTOS (dp6) are shown in a bar chart. **(J)** The dissociation constants (*K*_D_) of GmCERK1^ECD^ mutants with CSOS (dp6) are shown in a bar chart. **(K)** The dissociation constants (*K*_D_) of GmNFR5a^ECD^ mutants with CTOS (dp6) are shown in a bar chart. **(L)** The dissociation constants (*K*_D_) of GmNFR5a^ECD^ mutants with CSOS (dp6) are shown in a bar chart. **(M)** Predicted overall structure of GmCERK1^ECD^ in complex with CTOS (dp4) (left) or CSOS (dp4) (right). The GmCERK1^ECD^ structure is shown in pink. **(N)** Predicted overall structure of GmNFR5a^ECD^ in complex with CTOS (dp4) (left) or CSOS (dp4) (right). The GmNFR5a^ECD^ structure are shown in blue. **(O)** Predicted detailed model of hydrogen bonds and hydrophobic interaction between GmCERK1^ECD^ with CTOS (dp4) (left) or CSOS (dp4) (right). Black dots: carbon atoms, blue dots: nitrogen atoms, red dots: oxygen atoms. **(P)** Predicted detailed model of hydrogen bonds and hydrophobic interaction between GmNFR5a^ECD^ with CTOS (dp4) (left) or CSOS (dp4) (right). **(Q)** Sequence alignment of LysM2 in several LysMs‐containing RLKs and RLPs can predict potential critical residues for CTOS/CSOS binding. Consensus and similar amino acid residues for all sequences are highlighted in red and shown in blue boxes, respectively. acc, accessibility.

To further explore whether GmNFR5a and GmCERK1 bind to CTOS and/or CSOS, the ectodomains (ECD) of GmNFR5a and GmCERK1 were expressed in and purified from *Pichia pastoris* (Figure S8), and the binding ability of GmNFR5a^ECD^ and GmCERK1^ECD^ to CTOS and/or CSOS was then analyzed using microscale thermophoresis (MST). AtCERK1^ECD^ was used as a control and its dissociation constant (*K*_D_) with CTOS was 0.66 ± 0.27 mM (Figure S9). This result is consistent with a previous report by Cao et al. (2014), in which the binding affinity was measured using isothermal titration calorimetry (ITC), with a *K*_D_ of 0.455 mM for AtCERK1^ECD^ with CTOS (Cao et al., 2014). However, the *K*_D_ of GmCERK1^ECD^ and CTOS was 3.25 ± 0.95 mM (Figure 3A). The *K*_D_ of CSOS with AtCERK1^ECD^ and GmCERK1^ECD^ were 2.73 ± 0.43 mM and 10.59 ± 1.46 mM (Figures 3B, S9), respectively. The *K*_D_ of GmNFR5a^ECD^ with CTOS and CSOS were 20.54 ± 6.07 mM and 7.04 ± 2.62 mM (Figure 3E, F), respectively, suggesting that GmNFR5a^ECD^ has a higher affinity for binding to CSOS compared with CTOS. These results demonstrated that CTOS and CSOS bind directly to GmNFR5a and GmCERK1.

Previous studies have shown that chitin is recognized by the LysM2 domain of OsCEBiP or AtLYK5 (Cao et al., 2014; Liu et al., 2016). To determine the binding sites for CTOS and CSOS, we predicted the structures of the CTOS‐bound and CSOS‐bound LysM protein complexes using molecular docking. GmNFR5a^ECD^ and GmCERK1^ECD^ have three closely spaced tandem LysMs that form a conserved *β*–*α*–*α*–*β* structure (Figure 3M, N). Three‐dimensional structure prediction showed that the LysM2 domain of GmNFR5a^ECD^ can connect to CTOS (dp4) or CSOS (dp4) (Figure 3N). Similarly, CTOS (dp3) and CSOS (dp3) were also predicted to connect to the LysM2 domain of GmNFR5a^ECD^ (Figure S10B, C). In addition, the LysM2 domain of GmCERK1^ECD^ was predicted to bind to CTOS and CSOS (dp4 or dp3) (Figures 3M, S10D, E). Unexpectedly, despite extensive differences in the sequences of the extracellular LysM2 domains of GmNFR5a, AtLYK5, and OsCEBiP (Figure 3Q), the superposition of GmNFR5a^ECD^, AtLYK5^ECD^, and OsCEBiP^ECD^ revealed high structural similarity (Figure S10F). Chitin oligosaccharide (dp3) and CSOS (dp3) were predicted to interact with the LysM2 domain of GmNFR5a^ECD^, AtLYK5^ECD^, and OsCEBiP^ECD^, as revealed by the structural superposition (Figure S10G, H). This interaction could account for the observed variation in sequences of CTOS‐binding proteins across different plant species.

Molecular docking predictions of GmCERK1^ECD^ interacting with CTOS (dp4) and CSOS (dp4) also showed interactions between specific functional groups of the protein and the putative substrates. Within GmCERK1^ECD^, amino acids Asn136, Gln106, Glu110 and Tyr109 interacted with the acetyl groups on CTOS‐1, CTOS‐2, and CTOS‐4 of CTOS (dp4), correspondingly (Figure 3O). A hydrogen bond formed between the Asp135 methylol group of GmCERK1^ECD^ and the C2 methylol group of CTOS‐2 of CTOS (dp4). Additionally, the methylol and amino groups of Ile137 interacted with the amino group of CTOS‐2 and the C5 methylol group of CTOS‐3, respectively, of CTOS (dp4) (Figure 3O). The methylol groups of CSOS‐1 and CSOS‐3 of CSOS (dp4) were predicted to bind to the Asp139 hydroxyl group and the Asp107 methylol group of GmCERK1^ECD^, respectively (Figure 3O). Furthermore, Ile137 could simultaneously bind to the C3 hydroxyl and C4 amino groups of CSOS‐2 of CSOS (dp4). The C3 hydroxyl group of CSOS‐3 of CSOS (dp4) formed a hydrogen bond with the amide carbonyl oxygen of Gln106, while Asp135 and Glu110 interacted with the hydroxyl and amino groups of CSOS‐4 of CSOS (dp4) (Figure 3O).

To investigate the relevance of these specific amino acid residues of GmCERK1^ECD^ to CTOS and CSOS binding, we expressed and purified proteins with mutations of these residues in *P. pastoris* and obtained four mutant proteins: Y109R, P134H, I137R, and P138A (Figure S8). Compared with the control (WT), Y109R and I137R mutations impaired the binding activity of GmCERK1^ECD^ to CSOS (Figure 3C, D), but did not affect GmCERK1^ECD^ binding activity with CTOS (Figure 3A, I), suggesting the critical role of these two sites in CSOS binding. In addition, the P134H and P138A mutations significantly enhanced the binding activity of GmCERK1^ECD^ with CSOS (Figure 3B, J), but did not affect the interaction between GmCERK1^ECD^ and CTOS (Figure 3A, I). This suggests GmCERK1^ECD^ adopted specific binding sites to CSOS, and modulation of the binding sites might be expected to trigger a stronger immune response by CSOS.

In the predicted structures of GmNFR5a^ECD^ interacting with CTOS (dp4) and CSOS (dp4), a hydrogen bond was evident between the backbone nitrogen of Lys152 and the acetyl carbonyl oxygen of CTOS‐1 of CTOS (dp4), while the side‐chain nitrogen of Ile155 interacted with the methylol group of CTOS‐1 of CTOS (dp4) (Figure 3P). Cooperative hydrogen bonding occurred between the main‐chain nitrogen of Gly121 and the carbonyl oxygen of the acetyl group of CTOS‐2 of CTOS (dp4). The main‐chain hydroxyl and nitrogen of Asn151 bound with the C1 methylol group of CTOS‐2 of CTOS (dp4) and the CTOS‐3–CTOS‐4 glycosidic bond, respectively. Furthermore, the main‐chain hydroxyl group and side‐chain nitrogen of Leu153 formed hydrogen bonds with the nitrogen of CTOS‐2 and the C5 methylol group of CTOS‐3, respectively, of CTOS (dp4). The Pro150 residue interacted with the methylol group of CTOS‐3 and the C3 hydroxyl group of CTOS‐4 of CTOS (dp4), while the nitrogen of Tyr125 formed a hydrogen bond with the acetyl group of CTOS‐4 of CTOS (dp4) (Figure 3P). Additionally, the Asn151 amide carbonyl oxygen and the C3 hydroxyl of CSOS‐1 of CSOS (dp4) were involved in hydrogen bonding, with Ser123 binding to the amino group of CSOS‐1 of CSOS (dp4) (Figure 3P). The methylol group of Pro150 and the side‐chain nitrogen of Leu153 bound to the methylol group of CSOS‐2 of CSOS (dp4). Meanwhile, the methylol group of Leu153 and the amide carbonyl oxygen of Gln120 could hydrogen bond with the amino group of CSOS‐3 of CSOS (dp4) (Figure 3P).

For further elucidation, we generated five GmNFR5a^ECD^ mutations: S123T/F126T, F124R, P150H, L153R, and P154H (Figure S8). F124R and L153R impaired the binding activity of GmNFR5a^ECD^ to CSOS (Figure 3G, H), whereas these two mutations increased the binding activity of GmNFR5a^ECD^ to CTOS (Figure 3E, K), suggesting these two sites have different effects on GmNFR5a^ECD^ binding to CTOS and CSOS, and are necessary for binding CSOS. Additionally, S123T/F126T, P150H, and P154H mutations exhibited considerably enhanced CTOS‐ and CSOS‐binding activity within GmNFR5a^ECD^ (Figure 3E, F, K, L). These findings showed that both CTOS and CSOS bind to the LysM2 domain of GmNFR5a^ECD^. Taken together, we demonstrated direct binding of CTOS and CSOS to both GmNFR5a^ECD^ and GmCERK1^ECD^, and identified the key binding sites of GmCERK1^ECD^ and GmNFR5a^ECD^ with CSOS.

### Functional validation of residues essential for GmCERK1/GmNFR5a‐mediated plant immunity induced by CTOS/CSOS

**Figure 4:**
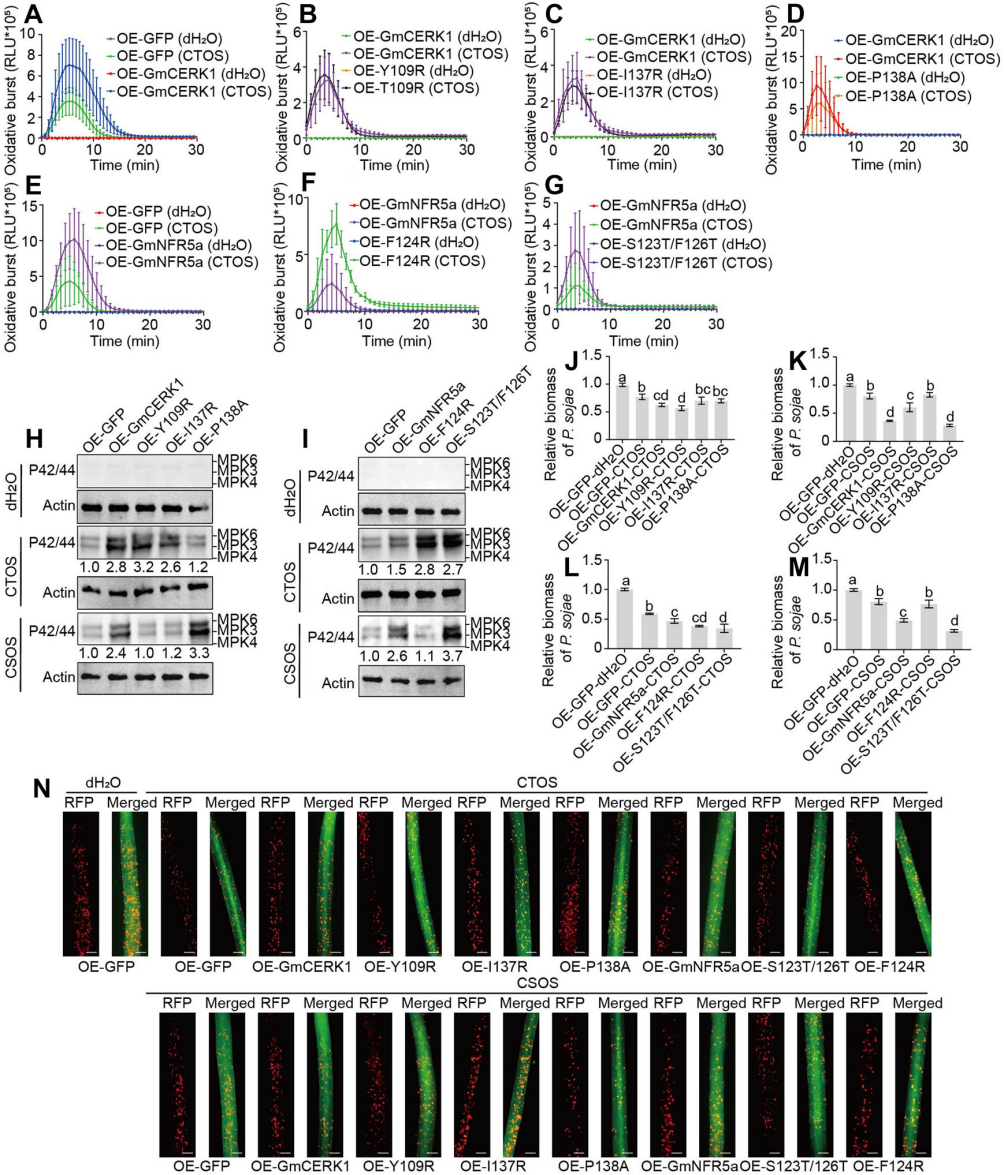
Functional validation of residues essential for GmCERK1/GmNFR5a‐mediated immunity triggered by CTOS/CSOS **(A–D)** Reactive oxygen species (ROS) production triggered by CTOS (50 mg/L) in soybean root hairs overexpressing *GmCERK1* **(A)** and its mutants *Y109R* **(B)**, *I137R* **(C)**, and *P138A* **(D)**. Mean RLU (relative luminescence unit) (± *SD*) are shown (*n* = 6). **(E–G)** Reactive oxygen species production triggered by CTOS (50 mg/L) in soybean root hairs overexpressing *GmNFR5a* **(E)** and its mutants *F124R* **(F)** and *S123T/F126T* **(G)**. Mean RLU (± *SD*) are shown (*n* = 6). **(H**, **I)** Chitin oligosaccharide/chitosan oligosaccharide triggered mitogen‐activated protein kinase (MAPK) phosphorylation in overexpressed‐*GmCERK1*, ‐*Y109R*, ‐*I137R*, ‐*P138A* **(H)** and overexpressed‐Gm*NFR5a*, ‐*F124R*, and ‐*S123T/F126T* **(I)** transgenic soybean root hairs at 10 min. Total protein was analyzed by immunoblot with an antibody for phosphorylated MPK6/3/4 (P42/44). The total band intensities were quantified using ImageJ software. Actin was used as an internal control to indicate the sample loading quantity. **(J**, **K)** The relative biomass of *Phytophthora sojae* in infected overexpressed‐*GmCERK1*, ‐Y109R, ‐I137R, and ‐P138A hairy roots treated by CTOS **(J)** or CSOS **(K)**, measured by genomic DNA quantitative polymerase chain reaction (qPCR) and normalized to the green fluorescent protein (GFP) control. **(L**, **M)** The relative biomass of *P. sojae* in infected overexpressed‐Gm*NFR5a*, ‐F124R, and ‐S123T/F126T hairy roots treated by CTOS **(L)** or CSOS **(M)**, measured by genomic DNA qPCR and normalized to the GFP control. **(N)** Expression levels of Gm*NFR5a*, Gm*CERK1*, and their mutants in soybean hairy roots affect CTOS/CSOS‐triggered defense against *P. sojae*. Transgenic hairy roots overexpressing *GmCERK1*, *Y109R*, *I137R*, *P138A*, Gm*NFR5a*, *F124R*, and *S123T/F126T* were inoculated with *P. sojae* zoospores expressing red fluorescent protein (RFP). OE‐*GFP* is used as a negative control. Oospore production at 48 h post inoculation (hpi) is shown. Six independent experiments gave similar results. Left, RFP; right, merge (RFP + GFP). Root hairs are treated with dH_2_O, CTOS, and CSOS, respectively. Scale bars, 0.2 mm. In **(J–M)**, different letters indicate a significant difference at the *P* < 0.05 level by Duncan's test. All experiments were repeated three times with similar results.

To validate the functional roles of GmCERK1/GmNFR5a binding sites in plant immunity induced by CTOS and CSOS, we overexpressed site‐specific mutants *in planta* to assess their effects on pathogen resistance. Based on MST assays and predictive analyses, GmCERK1 mutants (Y109R, P134H, I137R, P138A) and GmNFR5a mutants (G121P, Y125A, S123T/F126T, F124R, P150H, L153R, P154H) were selected for *in vivo* studies. We heterologously expressed *GmCERK1*, *GmNFR5a*, and their mutants in *N. benthamiana*. The results demonstrated that overexpression of the wild‐type (WT) GmCERK1 and GmNFR5a proteins significantly enhanced CTOS‐ and CSOS‐induced disease resistance in plants (Figure S11A, B). Compared with the WT GmCERK1 and GmNFR5a proteins, overexpression of the *I137R* and *S123T/F126T*, *F124R*, and *P150H* mutants further boosted CTOS‐triggered ROS burst (Figure S11A, B). While P138A in GmCERK1 enhanced CSOS‐induced MAPK activation, Y109R and I137R attenuated it (Figure S11C, D). Subsequent validation in soybean root hair overexpression lines (excluding P150H, which failed to express) demonstrated that *GmCERK1*/*GmNFR5a* overexpression potentiated both ROS burst and MAPK activation upon CTOS/CSOS treatment (Figure 4). Specifically, P138A in GmCERK1 reduced CTOS‐induced immunity (Figure 4D), whereas F124R and S123T/F126T in GmNFR5a enhanced it (Figure 4F, G). In addition, the Y109R and I137R in GmCERK1 and F124R in GmNFR5a significantly reduced CSOS‐induced MAPK activity (Figure 4H). In contrast, the P138A in GmCERK1 and S123T/F126T in GmNFR5a enhanced CSOS‐triggered MAPK activation (Figure 4I).

Notably, overexpression of *GmCERK1* and *GmNFR5a* significantly enhanced disease resistance in soybean induced by CTOS and CSOS (Figure 4J–N). However, the Y109R, I137R, and F124R mutants suppressed CSOS‐triggered disease resistance, whereas the S123T/F126T mutation potentiated resistance responses to both CTOS and CSOS. These findings demonstrate that Y109 and I137 in GmCERK1, along with F124 in GmNFR5a, serve as critical binding sites for CSOS recognition. Importantly, structural modifications at S123T/F126T in GmNFR5a significantly enhance CTOS‐ and CSOS‐induced disease resistance, providing molecular targets for improving soybean resistance through receptor engineering.

### GmNFR5a interacts with GmCERK1 in the presence of CTOS/CSOS

Given that both GmNFR5a and GmCERK1 are required for an effective response to CTOS or CSOS, we reasoned that the two proteins might form a heterodimer in the presence of CTOS/CSOS. To investigate this possibility, we analyzed the subcellular localization of GFP‐tagged GmCERK1 and GmNFR5a transiently expressed in *N. benthamiana*. The green fluorescence of GFP‐GmCERK1 and GFP‐GmNFR5a localized predominantly at the cell periphery (Figure S13A). Additionally, when GFP‐GmCERK1 and GFP‐GmNFR5a were co‐expressed with Remorin (StREM1.3), a known plasma membrane marker (Guo et al., 2019), co‐localization was observed, showing their plasma membrane localization (Figure S13B). This observation was consistent with the predicted transmembrane domains of GmCERK1 and GmNFR5a (Figure S10A).

**Figure 5:**
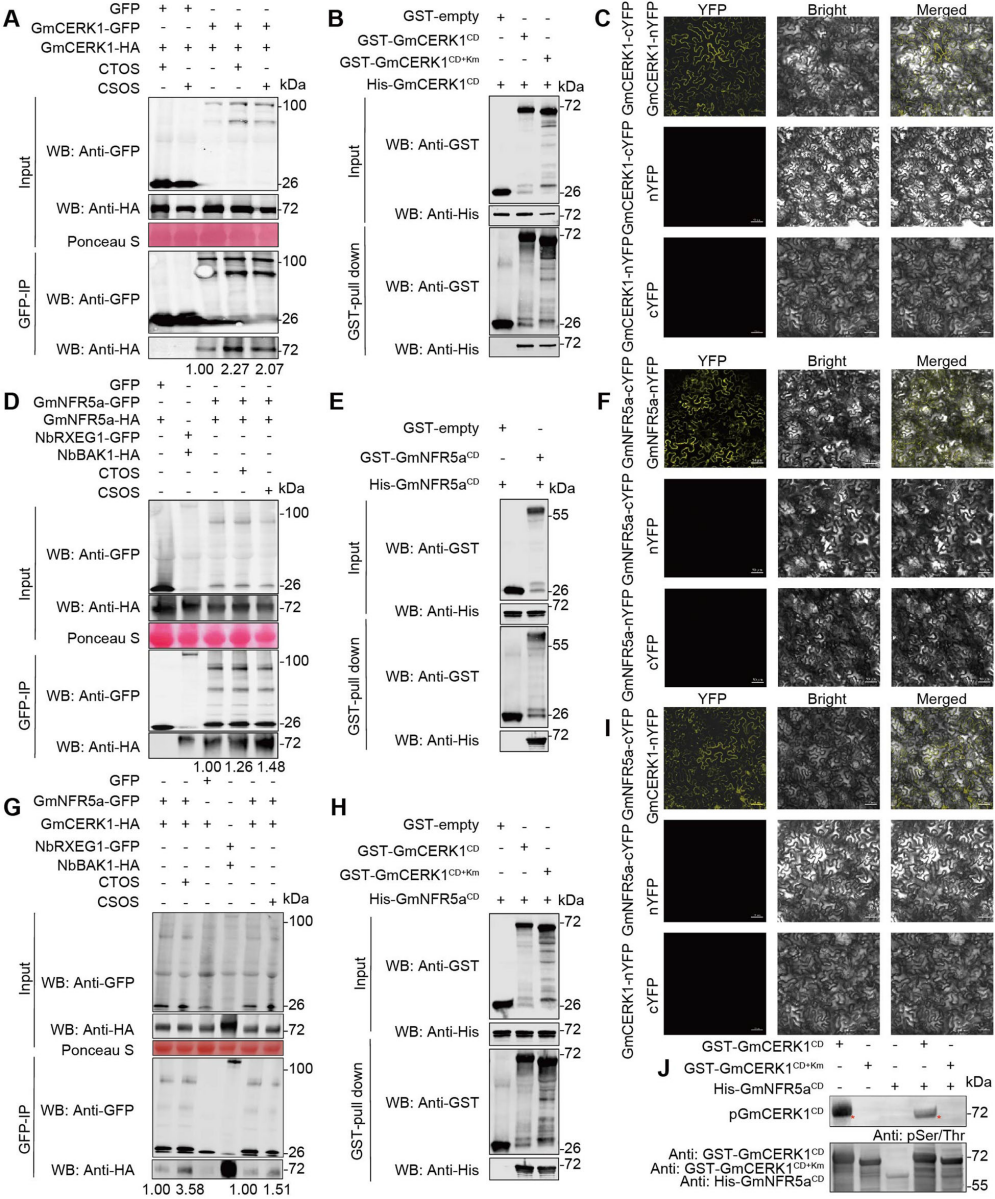
GmNFR5a and GmCERK1 form a heterotetramer in the presence of CTOS/CSOS **(A)** GmCERK1 forms a strong homodimer after CTOS/CSOS treatment. Green fluorescent protein (GFP)‐tagged GmCERK1 and HA‐tagged GmCERK1 were co‐expressed in *Nicotiana benthamiana* plants. The treated leaves with CTOS/CSOS (50 mg/L) for 5 min were used to extract proteins. Co‐immunoprecipitation was performed using anti‐HA antibody. Ponceau S: Ponceau staining indicates the RuBisCO protein. Molecular mass markers are shown (in kilodaltons). **(B)** The interaction between GST‐GmCERK1^CD^ and His‐GmCERK1^CD^ using GST pull‐down. Protein extracts of GST‐GmCERK1^CD^ and His‐GmCERK1^CD^, GST‐GmCERK1^CD+Km^ and His‐GmCERK1^CD^, or GST‐tag and His‐GmCERK1^CD^ were mixed and incubated with GST beads, respectively, followed by washing with PBS buffer five times. Proteins in the input samples and pull‐down samples were detected by western blot with anti‐GST and anti‐His antibodies. CD, cytoplasmic domain; Km, kinase inactivation mutant. **(C)** Illustration of the application of bimolecular fluorescence complementation (BiFC) technology to demonstrate the GmCERK1 forming a strong homodimer in plant cells. GmCERK1 fused with the C‐terminus of yellow fluorescent protein (YFP) was transiently expressed in *N. benthamiana* leaves with GmCERK1 fused to the N‐terminus of YFP. Co‐expression of GmCERK1‐cYFP with only the N‐terminus of YFP or GmCERK1‐nYFP with only the C‐terminus of YFP is used as a negative control. The interaction of GmCERK1‐cYFP and GmCERK1‐nYFP in the cytoplasm resulted in emission of a fluorescent signal. Scale bars: 10 μm. **(D)** GmNFR5a forms a strong homodimer after CTOS/CSOS treatment. GFP‐tagged GmNFR5a and HA‐tagged GmNFR5a were co‐expressed in *N. benthamiana* plants. The treated leaves with CTOS/CSOS (50 mg/L) for 5 min were used to extract proteins. Co‐immunoprecipitation was made using anti‐HA antibody. Ponceau S: Ponceau staining indicates the RuBisCO protein. Molecular mass markers are shown (in kilodaltons). **(E)** GST‐NFR5a^CD^ interacts with His‐NFR5a^CD^ *in vitro* by GST pull‐down assays. **(F)** Illustration of the application of BiFC technology to demonstrate the GmNFR5a forming a strong homodimer in plant cells. GmNFR5a fused with the C‐terminus of yellow fluorescent protein (YFP) was transiently expressed in *N. benthamiana* leaves with GmNFR5a fused to the N‐terminus of YFP. Co‐expression of GmNFR5a‐cYFP with only the N‐terminus of YFP or GmNFR5a‐nYFP with only the C‐terminus of YFP is used as a negative control. The interaction of GmNFR5a‐cYFP and GmNFR5a‐nYFP in the cytoplasm resulted in emission of a fluorescent signal. Scale bars: 10 μm. **(G)** GmNFR5a constitutively interacts with GmCERK1 *in vivo* by co‐immunoprecipitation (Co‐IP), dependent on CTOS/CSOS. The co‐expressed GmNFR5a‐GFP and GmCERK1‐HA in *N*. *benthamiana* leaves treated with CTOS/CSOS (50 mg/L) for 5 min were used to extract proteins. The interaction between NbRXEG1 and NbBAK1 is regarded as a positive control. **(H)** GST‐CERK1^CD^ or GST‐GmCERK1^CD+Km^ interacts with His‐NFR5a^CD^ *in vitro* by GST pull‐down assays. **(I)** GmCERK1 interacts with GmNFR5a in plant cells by BiFC. GmNFR5a fused with the C‐terminus of yellow fluorescent protein (YFP) was transiently expressed in *N. benthamiana* leaves with GmCERK1 fused to the N‐terminus of YFP. Co‐expression of GmNFR5a‐cYFP with only the N‐terminus of YFP or GmCERK1‐nYFP with only the C‐terminus of YFP is used as a negative control. The interaction of GmNFR5a‐cYFP and GmCERK1‐nYFP in the cytoplasm resulted in emission of a fluorescent signal. Scale bars: 10 μm. **(J)** *In vitro* kinase assay demonstrates that GmCERK1 can form auto‐phosphorylation but GmNFR5a cannot. The reaction mixtures were subjected to immunoblotting to detect substrate phosphorylation by using the anti‐pSer/Thr antibody. Red asterisk, auto‐phosphorylation band of GmCERK1.

Furthermore, we discovered that both GmCERK1 and GmNFR5a could form homodimers. Co‐immunoprecipitation (Co‐IP) assays demonstrated a strong interaction between GmCERK1‐GFP and GmCERK1‐HA in the presence of CTOS/CSOS (Figure 5A). *In vitro* pull‐down assays corroborated these findings, indicating that His‐tagged GmCERK1 cytoplasmic domain (CD) could be pulled down using GST‐fused GmCERK1^CD^ or the kinase‐inactive mutant form GST‐GmCERK1^CD+Km^, but not with GST alone (Figure 5B). Bimolecular fluorescence complementation (BiFC) assays suggested that GmCERK1‐cYFP could interact with GmCERK1‐nYFP at the cell membrane (Figure 5C). GmNFR5a also formed homodimers, as shown by Co‐IP, BiFC, and GST pull‐down assays (Figure 5D‐F).

Co‐immunoprecipitation assays indicated that GmNFR5a and GmCERK1 interact *in planta*. GmCERK1‐HA co‐immunoprecipitated with GmNFR5a‐GFP, but not with GFP alone. The known interaction between NbRXEG1 and NbBAK1 served as a positive control (Wang et al., 2018). All proteins were detectable in the input fractions (Figure 5G). These assays illustrated that GmNFR5a interacted strongly with GmCERK1 upon treatment with CTOS or CSOS. To further characterize the direct interaction between GmNFR5a and GmCERK1, we conducted *in vitro* pull‐down assays. The recombinant proteins GST‐GmCERK1^CD^, GST‐GmCERK1^CD+Km^, and His‐GmNFR5a^CD^ were expressed in and purified from *Escherichia coli*. The pull‐down assays detected His‐GmNFR5a^CD^ in complexes with GST‐GmCERK1^CD^ or GST‐GmCERK1^CD+Km^, but not with GST alone. This finding indicated that GmNFR5a directly interacted with GmCERK1 *in vitro*, a process that appears to be independent of the ATP‐binding site of GmCERK1 (Figure 5H). Furthermore, BiFC assays revealed that GmNFR5a‐cYFP can form a complex with GmCERK1‐nYFP at the cell membrane (Figure 5I).

*In vitro* phosphorylation assays showed that GmCERK1 is capable of auto‐phosphorylation, whereas GmNFR5a is not, with cross‐phosphorylation between the two proteins not apparent (Figure 5J). This indicates that GmNFR5a may act as an inactive RLK involved in chitooligosaccharide perception, while GmCERK1 plays a key role in signal transduction. Collectively, our data suggested a model in which GmNFR5a and GmCERK1 form a heterologous polymeric complex with chitooligosaccharides. GmNFR5a physically associates with GmCERK1, both *in vivo* and *in vitro*, and GmCERK1 is the principal agent for transducing the signal through its auto‐phosphorylation activity.

### GmCAK1 mediates CTOS/CSOS‐triggered immune responses and pathogen resistance in soybean

**Figure 6:**
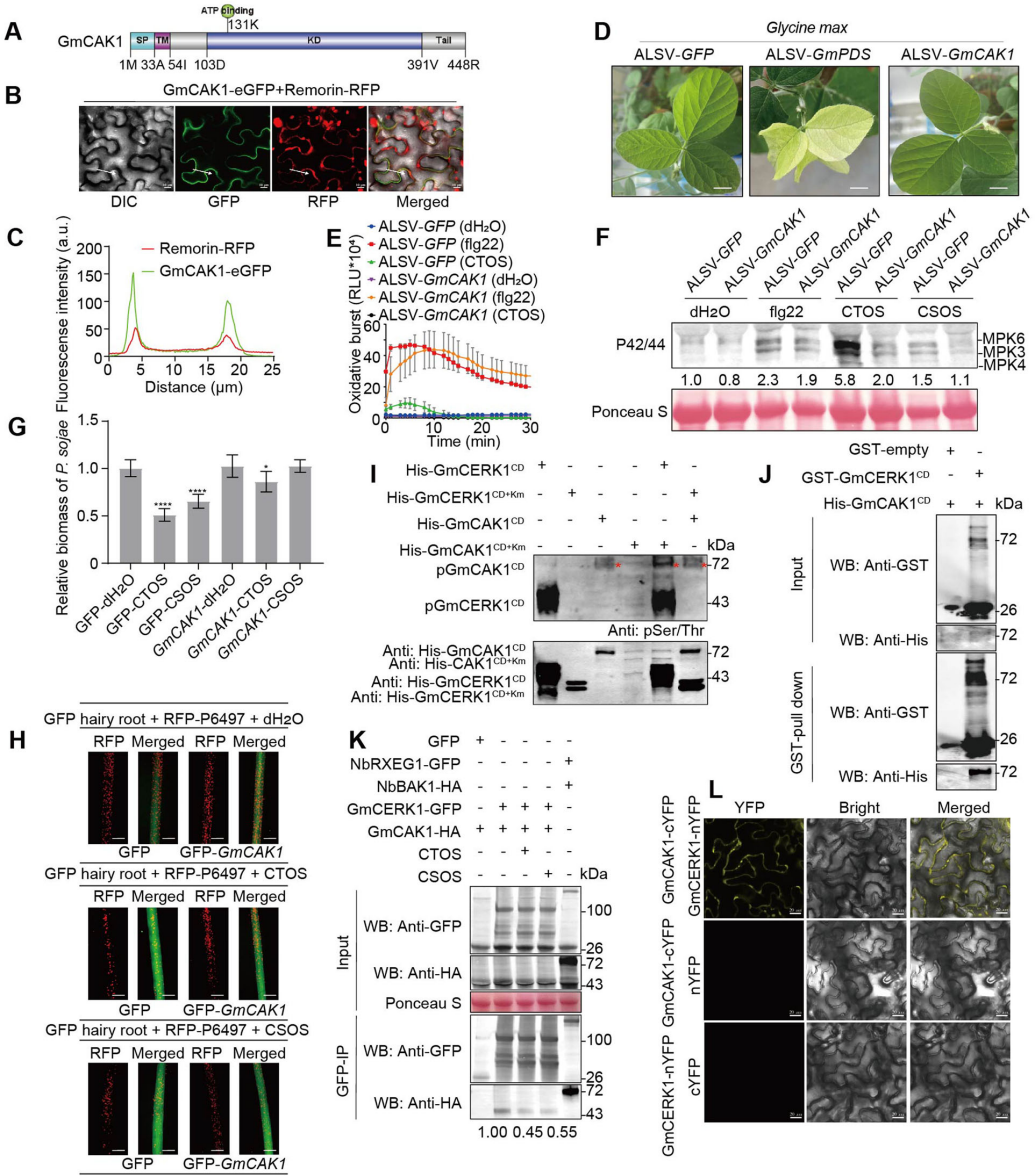
GmCAK1 functions in the CTOS/CSOS signaling pathway **(A)** Schematic representation of the domain architecture of GmCAK1. GmCAK1 contains a signal peptide (SP), a transmembrane domain (TM), a kinase domain (KD) containing an ATP‐binding site, and a C‐terminal domain (Tail), which are shown as different colors. **(B)** GmCAK1 localizes to the plasma membrane. Expression of GmCAK1‐eGFP fusion proteins in *Nicotiana benthamiana* through agro‐infiltration revealed that GmCAK1 is localized at the cell periphery. The Remorin protein as a membrane localization marker was used as a positive control. Fluorescence from epidermal cells in the infiltrated tissues was observed by confocal microscopy at 24 h post inoculation (hpi). Scale bars, 10 mm. **(C)** Fluorescence intensity profiles of GmCAK1‐eGFP with Remorin‐RFP in membrane transects (white arrowheads). *y*‐axis, green fluorescent protein (GFP) or red fluorescent protein (RFP) relative fluorescence intensity; *x*‐axis, transect length (mm). **(D)** Plant growth phenotype expressed recombinant apple latent spherical virus (ALSV)‐*GFP*, ALSV‐*GmPDS*, and ALSV‐*GmCAK1* in soybean Zhonghuang 13. After 20 d of inoculation of virus particles on soybean leaves, silenced‐*GmPDS* soybean plants show albinism. Scale bar: 1.5 cm. **(E)** Reactive oxygen species (ROS) production triggered by CTOS (50 mg/L) in leaf discs of soybean Zhonghuang 13 plants silenced the *GmCAK1* gene, flg22 as positive control. Mean RLU (Relative Luminescence Unit) (± *SD*) are shown (*n* = 6). **(F)** CSOS triggers mitogen‐activated protein kinase (MAPK) phosphorylation in silenced‐*GmCAK1* transgenic soybean leaf discs at 5 min, flg22 as positive control. Total protein was analyzed by immunoblot with an antibody for phosphorylated MPK6/3/4 (P42/44). The total band intensities were quantified using Image J. **(G)** The relative biomass of *Phytophthora sojae* in infected hairy roots treated by CTOS or CSOS, measured by genomic DNA quantitative polymerase chain reaction (qPCR) and normalized to the GFP control. Asterisks indicate statistically significant differences to control based on Student's *t‐*test (*P ≤ 0.05; ****P ≤ 0.0001). All experiments were repeated three times with similar results. **(H)** Expression level of Gm*CAK1* in soybean hairy roots affects CTOS/CSOS‐triggered defense against *P. sojae*. Transgenic hairy roots expressing GFP, silenced‐*GmCAK1* construct (GFP control or gene RNAi) were inoculated with *P. sojae* zoospores expressing red fluorescent protein (RFP). Oospore production at 48 h post inoculation (hpi) is shown. Six independent experiments gave similar results. Left, RFP; right, merge (RFP + GFP). From top to bottom, three lines of root hairs are treated with dH_2_O, CTOS, and CSOS, respectively. Scale bars, 0.2 mm. **(I)** *In vitro* kinase assay demonstrates that GmCERK1 phosphorylates GmCAK1. The reaction mixtures were subjected to immunoblotting to detect substrate phosphorylation by using the anti‐pSer/Thr antibody. To distinguish the size of GmCERK1^CD^ from GmCAK1^CD^, GmCERK1^CD^ and GmCERK1^CD+Km^ were cloned into the vector pET‐28a. Red asterisk, phosphorylation band of GmCAK1. **(J)** The interaction between GST‐GmCERK1^CD^ and His‐GmCAK1^CD^ by GST pull‐down. Protein extracts of GST‐GmCERK1^CD^ and His‐GmCAK1^CD^ were mixed and incubated with GST beads, respectively, followed by washing with PBS buffer five times. Proteins in the input samples and pull‐down samples were detected by western blot with anti‐GST and anti‐His antibodies. CD, cytoplasmic domain. **(K)** GmCERK1 interacts with GmCAK1 *in vivo* by co‐immunoprecipitation (Co‐IP). The co‐expressed GmCERK1‐GFP and GmCAK1‐HA in *N*. *benthamiana* leaves treated with CTOS/CSOS (50 mg/L) for 5 min were used to extract proteins. The interaction between NbRXEG1 and NbBAK1 is regarded as a positive control. **(L)** Illustration of the application of bimolecular fluorescence complementation (BiFC) technology to demonstrate the GmCERK1 interaction with GmCAK1 in plant cells. GmCAK1 fused with the C‐terminus of yellow fluorescent protein (YFP) was transiently expressed in *N. benthamiana* leaves with GmCERK1 fused to the N‐terminus of YFP. Co‐expression of GmCAK1‐cYFP with only the N‐terminus of YFP or GmCERK1‐nYFP with only the C‐terminus of YFP is used as a negative control. The interaction of GmCAK1‐cYFP and GmCERK1‐nYFP in the cytoplasm resulted in emission of a fluorescent signal. Scale bars: 10 μm.

We next investigated the intracellular signaling pathway that mediates CTOS/CSOS‐triggered immunity downstream of GmCERK1. To pinpoint the downstream target protein of GmCERK1 in soybean, we employed Co‐IP followed by liquid chromatography‐tandem mass spectrometry (LC‐MS/MS). A receptor‐like kinase protein, which we named GmCAK1 (CERK1‐Associated Kinase 1), co‐immunoprecipitated with GmCERK1 in all three biological replicates. The GmCAK1 protein is characterized by a signal peptide, a transmembrane domain, a kinase domain, and a C‐terminal tail (Figure 6A). Intriguingly, we discovered that GmCAK1 and Remorin colocalized at the plant cell plasma membrane (Figure 6B, C), indicating that GmCAK1 is a membrane‐localized protein lacking an extracellular domain. To further explore the role of GmCAK1 in soybean, we conducted ALSV‐induced gene silencing assays. Recombinant ALSV vectors expressing GmCAK1 were propagated in *N. benthamiana*, with ALSV vectors expressing GFP serving as a control. Following the yellowing of *N. benthamiana* leaves due to ALSV‐*GmCH42* expression, virus particles were harvested and inoculated onto the leaves of the soybean cultivar Zhonghuang 13 (Figure S6A). At 20 d post inoculation, soybean leaves silenced with ALSV‐*GmPDS* exhibited marked albinism, while silencing of GmCAK1 did not visibly impair soybean growth (Figure 6D). Compared with control plants harboring ALSV‐*GFP*, the *GmCAK1*‐silenced soybeans showed weaker CTOS/CSOS‐induced immune responses (Figure 6E, F). For instance, the activation of MAPK caused by CTOS (dp6) or CSOS (dp6) was obviously attenuated in soybeans harboring the ALSV‐*GmCAK1* silencing vector (Figure 6F). Additionally, the ROS burst normally triggered by CTOS (dp6) was entirely abolished in soybean harboring the ALSV‐*GmCAK1* silencing construct (Figure 6E).

To verify the contribution of GmCAK1 to the CTOS/CSOS‐induced resistance against *P. sojae*, we silenced *GmCAK1* via RNAi in soybean hairy roots (Figure 6H). In control hairy roots expressing GFP, treatment with CTOS (dp6) significantly bolstered soybean defense against *P. sojae* compared with treatment with dH_2_O, as evidenced by quantitative real‐time polymerase chain reaction (qRT‐PCR) analysis of the pathogen biomass (*P* < 0.0001; Figure 6G). However, this CTOS‐induced resistance was diminished in *GmCAK1*‐silenced hairy roots. Likewise, pathogen biomass analysis revealed no significant difference between the control (GFP‐dH_2_O) and *GmCAK1*‐silenced soybean treated with CSOS (dp6) (Figure 6G). Collectively, these results showed that GmCAK1 plays a positive role in CTOS/CSOS‐mediated soybean resistance to *P. sojae*, probably as a component of the intracellular signal transduction pathway initiated by these signals.

### GmCERK1 interacts with and phosphorylates GmCAK1

To further elucidate the contribution of GmCERK1 to downstream signaling in the CTOS (dp6) or CSOS (dp6) response pathway, we investigated the interaction between GmCERK1 and GmCAK1. Co‐immunoprecipitation assays demonstrated an association between GmCERK1‐GFP and GmCAK1‐HA *in planta*, with GFP alone serving as a negative control (Figure 6K). Notably, the interaction between GmCERK1 and GmCAK1 was diminished in the presence of CTOS (dp6) or CSOS (dp6). This suggests that the propagation of downstream phosphorylation signals involves dissociation of GmCAK1 from GmCERK1. Subsequent *in vitro* pull‐down assays corroborated the physical interaction between GmCERK1 and GmCAK1 (Figure 6J). GST‐GmCERK1^CD^ and His‐GmCAK1^CD^ were expressed in and purified from *E. coli*. In the pull‐down assays, His‐GmCAK1^CD^ formed complexes with GST‐GmCERK1^CD^ but not the GST‐only controls, indicating that GmCERK1 interacted with GmCAK1 *in vitro*. Bimolecular fluorescence complementation assays revealed that GmCAK1‐cYFP interacted with GmCERK1‐nYFP at the cell membrane (Figure 6L). Crucially, *in vitro* phosphorylation assays showed that GmCAK1^CD^ possessed auto‐phosphorylation activity (Figure 6I). Additionally, we observed that GmCERK1^CD^ could phosphorylate GmCAK1^CD^, whereas GmCAK1^CD^ was incapable of phosphorylating GmCERK1^CD^ (Figure 6I). These results further supported the notion that GmCAK1 acts downstream of GmCERK1 and plays an integral role in transmitting signals initiated by GmCERK1.

## DISCUSSION

Chitin and its oligosaccharides (CTOS) are well known PAMPs that activate characteristic immune responses in Arabidopsis, rice, and several other plant species (Cao et al., 2014; Lee et al., 2014; Liu et al., 2016; Brulé et al., 2019). Nonetheless, the details of CTOS signaling in soybean remain largely unknown. Chitosan, a deacetylated derivative of chitin, and its oligosaccharides (CSOS), trigger plant disease resistance and hold substantial promise as a natural compound for fungal control (Gubaeva et al., 2018; Lopez‐Moya et al., 2021), yet the specifics of its action in plants have yet to be fully elucidated. In this study, we established that GmNRF5a and GmCERK1, located in the plasma membrane of soybean cells, are instrumental for mediating CTOS and CSOS elicitor signals for defense responses. Notably, CTOS and CSOS bind directly to both GmNRF5a and GmCERK1. However, these proteins exhibit distinct binding modalities with the two elicitor molecules. Importantly, we have identified the key binding sites of GmCERK1 and GmNFR5a with CSOS, and these binding activities are different from those of CTOS. In addition, GmCERK1 has a higher binding affinity for CTOS than for CSOS (Figure 3A, B). Silencing of *GmCERK1* completely abolished the immunity induced by CTOS, while the immunity induced by CSOS was only partially diminished. This led us to propose that GmCERK1 plays a more important role in the immunity signaling cascade triggered by CTOS than in that triggered by CSOS. Furthermore, both CTOS and CSOS share a common downstream transmitter in the form of the receptor‐like kinase GmCAK1. These findings implied that CTOS and CSOS evoke similar and overlapping PAMP signaling pathways within soybean.

Chitin oligosaccharide binds specifically to both GmNFR5a and GmCERK1 (Figure 3A, E). The intensity of CTOS binding to GmCERK1 is significantly lower than that of its binding to AtCERK1. The binding affinity with CTOS or CSOS may link to the evolution of CERK1 to fulfill the multifunctionality in leguminous plants. CERK1 and NFR5 not only play crucial roles in plant immunity but also participate in plant symbiosis (Broghammer et al., 2012; Feng et al., 2019; Zhang et al., 2021; Wang et al., 2024). NFR5, designated as NFP in *M. truncatula*, is a pivotal LysM‐RLK that mediates plant responses to microbial lipochitooligosaccharides (LCOs) and chitooligosaccharides (Bozsoki et al., 2017; Rey et al., 2019). It participates in both symbiotic and immune signaling pathways, with distinct species‐specific characteristics. Although GmNFR5a lacks kinase activity, its binding to CTOS suggests it may form a CTOS‐inducible complex with GmCERK1 to activate soybean immunity. These observations parallel the mechanisms observed in Arabidopsis and rice (Shimizu et al., 2010; Cao et al., 2014). Chitin oligosaccharide binding leads to the formation of an AtCERK1–AtLYK5 complex essential for the phosphorylation of AtCERK1 (Petutschnig et al., 2010; Cao et al., 2014). Similarly, prior studies in rice indicated that OsCEBiP can bind directly to CTOS via its central LysM domain (LysM2) (Shimizu et al., 2010; Liu et al., 2016), while OsCERK1 acts as a co‐receptor, disseminating signals via its Ser/Thr kinase activity. In both instances, CERK1 possesses a functioning intracellular kinase domain and forms a complex with another LysM‐RLK—GmNFR5a/AtLYK5/OsCEBiP—that lacks this kinase activity. The binding affinity between CTOS and GmNFR5a is slightly lower than that of GmCERK1. We therefore speculate that there may be other proteins in soybean primarily responsible for recognizing CTOS (Figure S17).

These commonalities point to an evolutionarily well conserved mechanism for CTOS signaling in plants. Despite the low sequence similarity between GmNFR5a and AtLYK5 (Figure S14), and considerable dissimilarity in their extracellular LysM2 domain sequences (Figure 3Q), the crucial residues for CTOS binding are positioned within conserved areas of the secondary structures (Figure 3P, Q). Structural overlays of these proteins disclose a universally conserved mechanism for CTOS detection by LysM proteins (Figure S10F, G), potentially explaining the wide variation in CTOS signaling in different plants. Furthermore, S123T/F126T mutations in GmNFR5a^ECD^ significantly enhanced CTOS‐binding activity (Figure 3E, K), thereby potentiating CTOS‐induced disease resistance in soybean (Figure 4L). This structural modification underscores the functional significance of these residues in mediating pathogen recognition and immune activation. Collectively, our findings elucidate the molecular intricacies underlying the interactions of GmNFR5a with CTOS, enhancing our understanding of the role of this protein in soybean immunity.

Protein dimerization followed by subsequent phosphorylation are typical steps of ligand‐mediated immune activation. In Arabidopsis, AtBRI1 homodimerizes or heterodimerizes with AtBAK1 (Wang et al., 2005; Santiago et al., 2013). In both rice (Hayafune et al., 2014) and Arabidopsis (Cao et al., 2014), the CTOS receptor complex operates as a heterotetramer. Within this configuration, OsCEBiP binds a single CTOS molecule from opposing sides, leading to OsCEBiP dimerization (Hayafune et al., 2014). Together, our diverse chemical and biological assays revealed that ligand‐induced homodimerization of GmCERK1 and GmNFR5a occurs in the presence of biologically active CTOS (Figure 5A, D). Nevertheless, the intricacies of the underlying molecular mechanisms promoting dimerization remain subjects for future investigation. Additionally, GmNFR5a physically interacted with GmCERK1 both *in vivo* and *in vitro* (Figure 5G–I). We hypothesize that, in the presence of CTOS, GmNFR5a and GmCERK1 form a heterologous polymeric complex, with dimerization of GmNFR5a bringing the associated GmCERK1 molecules into proximity and thereby triggering the auto‐phosphorylation of GmCERK1 (Figure 5J).

In contrast with CTOS, the CSOS‐induced immune signal transduction remains unclear. GmNFR5a is the first identified immune sector regulating CSOS signaling. Notably, residue F124 in GmNFR5a serves as a critical binding site for CSOS, but this residue does not affect CTOS‐induced disease resistance (Figure 4), indicating that GmNFR5a interaction with CSOS differs from that of CTOS with GmNFR5a. In addition, our study identified Y109 and I137 as key residues in GmCERK1 for CSOS binding. However, Y109R and I137R mutations in GmCERK1 did not disrupt CTOS binding; this discrepancy may arise from differences in the properties of the substituted amino acids. Additionally, our gene silencing experiments implicated GmCERK1 in the CSOS signaling pathway in soybean (Figures 2D, F, H, S6C), highlighting the vital role of GmCERK1 in CSOS signal transduction. The plant resistance elicited by CSOS was somewhat weaker than that triggered by CTOS (Figure 1E, F), primarily because CSOS (dp4–6) failed to induce ROS production (Figure S3). Nonetheless, CSOS can induce MAPK activity in soybean, paralleling observations in Arabidopsis (Brulé et al., 2019). As essential signaling elements, MAPKs are critical for regulating diverse immune responses. The induction of MAPKs and the expression of defense genes in soybean cells treated with CSOS suggest the existence of corresponding PRRs in soybean. Because CSOS is an immune activator and can be mass produced from crab shell waste, it has great potential as a commercially viable and eco‐friendly option for use in crop protection (Kumar, 2000; Yin et al., 2010). Future research in soybean will focus on modifying GmNFR5a and/or GmCERK1 to bolster CSOS‐induced resistance. The identification of GmNFR5a as a plant immune sector for CSOS substantially broadens the spectrum of potential ligands for plant LysM‐domain‐containing proteins, advances our understanding of the immune activation mechanisms of CSOS, and fosters the broader application of this eco‐friendly agricultural product.

Our observations demonstrated the vital function of the soybean receptor‐like kinase GmCAK1 (CERK1‐associated kinase 1) in the transmission of GmCERK1‐mediated CTOS/CSOS signaling. Although GmCAK1 is a membrane‐localized protein (Figure 6B), it lacks an extracellular domain. GmCERK1 not only interacts with GmCAK1 but also phosphorylates it, implying an essential role for GmCAK1 in mediating downstream responses within the CTOS/CSOS signaling pathway. Additionally, the genetic sequence of CAK1 is notably conserved across various plant species (Figures S15, S16), leading us to surmise that CAK1 may be a universal player in the transmission of CERK1‐initiated signals to mediators of early signaling events in plants. Interestingly, the CAK1 protein is distantly related to the receptor‐complex‐associated RLCKs (such as OsRLCK176 and AtPBL27) (Figure S15), indicating that CAK1 is a new element that participates in CERK1 signaling. Previous studies have highlighted the phosphorylation of OsRLCK176 or AtPBL27 as a critical aspect of CERK1 signaling. This phosphorylation prompts the disengagement and activation of RLCKs (OsRLCK176/AtPBL27) from the CERK1 complex, subsequently triggering downstream substrate interactions (Ao et al., 2014; Yamada et al., 2016). The next question is whether there is a signaling pathway between RLCKs and CAK1. Notably, the RLCK family seems to serve as the connection between diverse PRRs and MAPK cascades, potentially acting as the link between PRRs and MAPKKKs. This led to the hypothesis that RLCKs have a critical influence on the activation of MAPK cascades in response to myriad extracellular signals (Wang et al., 2017). For instance, OsRLCK185 mediates immune signaling from the chitin receptor OsCERK1 to a MAPK signaling cascade by interfacing with a MAPK kinase kinase (Wang et al., 2017). *In vitro*, AtPBL27 phosphorylates MAPKKK5, an action that is reinforced by CERK1‐mediated phosphorylation of PBL27 (Yamada et al., 2016). Chitin oligosaccharide/CSOS‐induced MAPK activity implies the presence of a pivotal element downstream of GmCAK1 capable of setting off the MAPK cascade, and which transmits signals from GmCAK1. The precise mechanism by which GmCAK1 activates the MAPK cascade poses a fascinating question and calls for intensified scrutiny.

In summary, we propose a conceptual framework wherein CTOS/CSOS signaling is activated by the GmNFR5a–GmCERK1 complex. Although the binding mechanisms for CTOS and CSOS differ somewhat, both GmNFR5a and GmCERK1 can form CTOS/CSOS‐induced heteropolymers. This cooperative interaction facilitates the joint transduction of signals downstream through GmCAK1 (Figure S17). Our findings also allude to a phospho‐signaling pathway that connects the cell surface binding of CTOS/CSOS to the internal activation of GmCAK1 in soybeans (Figures 6I, S17).

## MATERIALS AND METHODS

### Plant materials and growth conditions

*Nicotiana benthamiana* plants were routinely maintained in climate chambers at 19°C–22°C for 4–6 weeks with a 14 h light/10 h dark photoperiod and LED lamps with a light intensity of ∼120–150 μmol m^−2^ s^−1^. Soybean (*Glycine max*) (cultivar Williams and Zhonghuang13) plants were routinely maintained in climate chambers at 25°C for ∼10 d under a 12 h light/12 h dark photoperiod and LED lamps with a light intensity of ∼120–150 μmol m^−2^ s^−1^.

### Preparation and characterization of chitooligosaccharides

Chitosan oligosaccharides were obtained by the enzyme preparation method as previously described (Zhang et al., 1999) from chitosan (≥ 95% (deacetylated), Cat. No. C105799; Aladdin). Chitin oligosaccharides were prepared by the *N*‐acetylation of CSOS (Xu et al., 2017). These two kinds of oligosaccharides were separated to different monomers by semi‐preparative HILIC chromatography. Nonlinear gradient was used for the “click” Xamide column (250 × 10.0 mm, 5 μm) with acetonitrile as mobile phase A, and ammonium formate solution (50 mM, pH 3.0) as mobile phase B. The flow rate was 3 mL/min. Under optimized eluent conditions, the different CTOS/CSOS monomer fractions were separated and collected for further experiments. Electrospray ionization mass spectrometry was performed to characterize the CTOS/CSOS monomers.

### Bioinformatic analysis

For the identification of the LysM gene family, we searched for LysM proteins in *Arabidopsis thaliana*, *Glycine max*, *Oryza sativa*, and *Zea mays*, using the HMMER and Pfam databases. The LysM model (PF01476) was obtained from the Pfam database to search for LysM family members in these plants. The protein sequences were obtained from the JGI Phytozome database. SignalP 6.0 and DeepTMHMM were used to predict the signal peptides and transmembrane domains in the identified LysM proteins. We used NCBI CD search to predict the protein kinase domains in the identified LysM proteins. For alignment and phylogenetic analysis, the protein sequences were aligned using Muscle and the alignment was trimmed using trimAI. The best‐fit model of protein evolution was evaluated using ModelFinder according to the Bayesian information criterion (BIC). A maximum likelihood phylogenetic tree was constructed using IQ‐TREE with UltraFast Bootstrap analysis of 1,000 replicates. The final tree was visualized using iTOL. The predicted structures of GmNFR5a and GmCERK1 were obtained from the AlphaFold Protein Structure Database (https://alphafold.ebi.ac.uk/) with the entries of AF‐A5YJV9‐F1 and AF‐A5YJV9‐F1, respectively. For oligosaccharide docking studies, 3D molecular structures of DP4 CTOS and CSOS were prepared using the carbohydrate builder module in the GLYCAM‐Web server (https://glycam.org/cb/). For protein binding analysis, the binding of GmNFR5a and AtLYK5 to chitin was studied using the 3D molecular structure of OsCEBiP complex (PDB ID: 5JCE) obtained from the Protein Data Bank. The simulations were performed using AutoDock Vina with an exhaustiveness of 36. The binding residues were visualized using PyMOL and LigPlot.

### Plasmid constructs

The silenced fragments *GmLysMs* or *GmCAK1* were amplified from cDNA of soybean (Zhonghuang 13 cultivar) leaves using PrimeSTAR GXL DNA Polymerase (Vazyme Biotech Co. Ltd, Nanjing, China) with the primers listed in Supplemental Data Table S1, and cloned into ALSV2 vector (Dong et al., 2022) for virus‐induced gene silencing in soybean. For Co‐IP and the protein localization assays, the *GmCERK1*, *GmNFR5a*, or *GmCAK1* gene was cloned into the vector pGR107‐3HA (Chen et al., 2023), and the *GmCERK1*, *GmNFR5a*, or *GmCAK1* gene was cloned into the vector pBin‐eGFP (Chen et al., 2023). For silencing in hairy roots, *GmCERK1*, *GmNFR5a*, and *GmCAK1* genes were cloned into the vector pFGC5941 (Qiu et al., 2023) for transformation. For *in vitro* GST pull‐down, *GmCERK1* ^*CD*^, *GmCERK1* ^*CD+Km*^, and *GmNFR5a* ^*CD*^ were cloned into the vector pGEX‐4T‐2 (Liu et al., 2023), and *GmCERK1* ^*CD*^, *GmNFR5a* ^*CD*^, *GmCAK1* ^*CD*^, and *GmCAK1* ^*CD+Km*^ were cloned into the vector pET‐32a (Liu et al., 2023). For the *in vitro* kinase assay, *GmCERK1* ^*CD*^ and *GmCERK1* ^*CD+Km*^ were cloned into the vector pET‐28a (Ma et al., 2020). For the BiFC assays, the coding region of *GmCERK1*, *GmNFR5a*, or *GmCAK1* was inserted into the vector pSPYCE (Walter et al., 2004), and the coding region of *GmCERK1* or *GmNFR5a* was inserted into the vector pSPYNE (Walter et al., 2004).

### Chitooligosaccharides‐induced immune assays

To measure the oxidation burst, leaf discs (Ø 0.5 cm) collected from 2‐week‐old soybean (cultivar Williams) were floated overnight in 200 μL sterile H_2_O in a 96‐well plate. The H_2_O was replaced with 200 μL reaction buffer containing luminol/peroxidase (35.4 mg/mL luminol, 10 mg/mL peroxidase) and chitooligosaccharides treatments (50 mg/L). flg22 was used as a positive control. The luminescence was measured using a GLOMAX96 microplate luminometer (Promega, Madison, WI, USA). The experiments for MAPK activation were carried out as described previously (Shinya et al., 2014). The MAPK activation was determined by immunoblots with anti‐phospho‐p44/42 MAPK antibody (#4370; Cell Signaling, Boston, MA, USA).

### Apple latent spherical virus‐induced gene silencing in soybean

Apple latent spherical virus‐induced gene silencing in soybean was performed as previously described (Li et al., 2000; Naitow et al., 2020; Dong et al., 2022). Plasmids pALSV1 (Li et al., 2000; Dong et al., 2022), pALSV2 (Li et al., 2000; Dong et al., 2022), pALSV2‐CH42 (Dong et al., 2022), pALSV2‐GmPDS (Dong et al., 2022), pALSV2‐GmLysMs, and pALSV2‐GmCAK1 were transformed into *Agrobacterium tumefaciens* GV3101. Mixed agrobacteria carrying pALSV1 with pALSV2, pALSV2‐GmPDS, pALSV2‐CH42, pALSV2‐GmPDS, pALSV2‐GmLysMs, or pALSV2‐GmCAK1 in equal proportions were injected into the six to eight leaf stage *N. benthamiana*. Viruses composed of pALSV1 and pALSV2 were named ALSV, viruses composed of pALSV1 and pALSV2‐AtCH42 were named ALSV: CH42, and so on. At 17 d after *Agrobacterium* infiltration, 0.4 g of the infected leaf was ground into powder. Then, 800 μL of 0.01 mol/L pH 7.5 PB buffer solution (per 1,000 mL PB buffer contains 16 mL 0.2 mol/L NaH_2_PO_4_ and 84 mL 0.2 mol/L Na_2_HPO_4_) was added and thoroughly mixed before incubating on ice for 30 min. The mixture was centrifuged at 4°C at 8,500 rpm for 10 min, followed by the addition of 40% PEG/NaCl solution (per 100 μL supernatant, 23 μL of 40% PEG/NaCl), which was then incubated on ice for 1 h. After centrifugation at 4°C, 13,000 rpm for 15 min, the supernatant was removed, and the remaining suspension was precipitated using 100 μL of 0.01 mol/L pH 7.0 PB buffer to enrich virus particles. The concentration of virus particles was measured through OD_260_ and adjusted to 2 μg/μL. Leaves of 8–10‐d‐old soybean (Zhonghuang 13) seedlings were inoculated with 20 μL of extracted virus particles. When ALSV‐GmPDS plants exhibited photo‐bleaching symptoms (∼20 d after inoculation with viruses), the silencing efficiency was detected by qRT‐PCR.

### Soybean hairy‐root transformation

Soybean (cultivar Williams) seeds were surface sterilized and germinated as previously described (Kereszt et al., 2007). Soybean cotyledons were removed from 10‐d‐old seedlings grown in vermiculite. Cotyledons were harvested at Day 7 by gently twisting them off the hypocotyl. Only unblemished cotyledons were employed for all protocols. Individual cotyledons were surface sterilized by wiping with an alcohol swab soaked in 70% ethanol. The alcohol swab was wrung out slightly before use, so that it was wet but not dripping. The surface‐sterilized cotyledon was then cut by making a small, roughly circular (0.4 cm diameter) cut about 0.3 cm from the petiole end of the cotyledon to inoculate with *Agrobacterium rhizogenes* (strain K599) cell suspensions. Before inoculation, the cells were centrifuged at 2,500 *g* in a tabletop centrifuge for 20 min or until a relatively tight pellet of the bacteria was obtained. The K599 pellets were drained briefly and then gently resuspended in 10 mM MgCl_2_ to a final OD_600_ of ∼0.4 for inoculation of cotyledon tissues. Inoculated cotyledons were placed in sterile Petri dishes containing MS medium and incubated in a growth chamber at 22°C with a 16‐h photoperiod. Hairy roots were monitored for green fluorescence production over a period of 4 weeks. For silencing in hairy roots, the transcript levels of the targeted gene and its closest paralogs were measured in fluorescent green hairy roots using qRT‐PCR to assess silencing efficiency.

### Purification of GmCERK1 and GmNFR5a ectodomains

The ectodomains of *GmCERK1*, *GmNFR5a*, and mutants were cloned into the yeast expression vector pPICZ*α*A (Thermo Fisher Scientific, Shanghai, China) through the double digestion and ligation using the restriction endonuclease *Eco*RI/*Xba*I (NEB, Suzhou, China) and T4 ligase. Then, the linearized recombinant plasmid pPICZ*α*A‐GmCERK1 and pPICZ*α*A‐GmNFR5a were transformed into *P. pastoris* X‐33. The positive colonies were selected on YPDS medium (1% w/v yeast extract, 2% w/v peptone, 2% w/v glucose, 2% w/v agar, 100 μg/mL antibiotic Zeocin (Thermo Fisher Scientific)) plates and verified by direct DNA sequencing. Protein expression was carried out initially in BMGY media (100 mM potassium phosphate pH 6.0, 1.34% w/v YNB, 4 × 10^−5^% w/v biotin, 1% v/v glycerol) at 28°C with shaking (180 rpm) until the OD_600_ reached 2.0–6.0. The cell pellet was transferred into BMMY medium (100 mM potassium phosphate pH 6.0, 1.34% w/v YNB, 4 × 10^−5^% w/v biotin, 0.5% v/v methanol) and continuously supplied with the addition of 0.5% (v/v) methanol every day for 5 d. Finally, the supernatant of the broth was concentrated and subjected to buffer change (buffer A: 40 mM Tris‐HCl pH 8.0, 200 mM NaCl) using Amicon stirred cells (Merck Ltd, Shanghai, China) equipped with 10 kDa cutoff ultrafiltration membranes. The obtained crude protein solution was applied to a Ni‐NTA column equilibrated with buffer A. The target protein was eluted by a stepwise imidazole gradient with increasing concentrations in buffer A. The eluted fraction was collected and desalted with repeated concentration and dilution using an Amicon Ultra centrifugal filter unit (EMD Millipore, Shanghai, China). The protein sample was further purified by an anion‐exchange chromatography with a HiTrap Q FF column and size exclusion chromatography with a HiLoad Superdex 200pg column. As for the mutants of GmCERK1^ECD^ and GmNFR5a^ECD^, their encoded genes were synthesized by Genewiz, Inc. Co. with the mutations introduced in the desired positions. The obtained recombinant pPICZ*α*A (Thermo Fisher Scientific) plasmids bearing the mutated positions were transformed into *P. pastoris* X‐33 cells, and expressed and purified similarly to their original proteins. In addition, the gene encoding the ectodomain of AtCERK1 (aa25–224) was also synthesized by Genewiz Inc. and overexpressed in *P. pastoris* X‐33 through the pPICZ*α*A vector (Thermo Fisher Scientific). Purification of AtCERK1^ECD^ was the same as described above. Preparation of the negative control UGT76G1 was performed (Yang et al., 2019).

### Microscale thermophoresis assay

GmCERK1^ECD^, GmNFR5a^ECD^, and all mutants were labeled with a fluorescent dye using the Monolith Protein Labeling Kit RED‐NHS second Generation (MO‐L011). The labeled protein (0.02 μM) was mixed with ligand diluted to appropriate serial concentrations in buffer containing 20 mM HEPES pH 7.0, 50 mM NaCl. After incubation at room temperature for 30 min, the mixtures were loaded into Monolith Capillaries (MO‐K022). Measurements were performed with a Monolith NT.115 instrument (Nanotemper Technologies GmbH, Beijing, China) (Huang and Zhang, 2021). The data were analyzed and fitted by plotting ligand concentrations against bound fractions using the MO.Affinity Analysis v3.0.5 software. The curve fitting figures were prepared using GraphPad Prism software. The assays were repeated three times to ensure reproducibility.

### Co‐immunoprecipitation assays

*Nicotiana benthamiana* leaves were harvested 48 h post inoculation (hpi) after agroinfiltration. Samples were ground in liquid nitrogen and incubated for 30 min in a lysis buffer (50 mM Tris‐HCl pH 8.0, 150 mM NaCl, 1.0% (v/v) NP‐40, 0.1% SDS (w/v) and 0.1% protease inhibitor cocktail (P9599; Sigma, St. Louis, MI, USA)). Supernatant was collected by centrifugation at 21,000 *g* for 15 min and then incubated with GFP‐trap beads (item No.: gta‐20; Chromotek, Hauppauge, NY, USA) at 4°C for 2 h. The GFP‐trap beads were pelleted by centrifugation at 500 *g* for 2 min and washed with lysis buffer at least four times. Proteins were eluted by boiling the beads in a protein loading buffer for 10 min and detected by immunoblot analysis with anti‐GFP (Cat No. M20004; Abmart, Shanghai, China) or anti‐HA (Cat No. M20013; Abmart, Shanghai, China).

### Bimolecular fluorescence complementation assays

*Agrobacterium tumefaciens* strain GV3101 was transformed with pSPYCE‐GmCERK1, pSPYCE‐GmNFR5a, pSPYCE‐GmCAK1, pSPYNE‐GmCERK1, or pSPYNE‐GmNFR5a plasmids, respectively, and then used for infiltration of the leaves of 3–4‐week‐old *N. benthamiana* plants. Fluorescence was visualized in the infiltrated area of the leaves at 48 hpi.

### *In vitro* GST pull‐down

Pull‐down assays were carried out using a ProFound Pull‐Down GST Protein–Protein Interaction Kit (Pierce) (GST, glutathione *S*‐transferase). *Escherichia coli* strain BL21 was used to express GST, GST‐GmCERK1^CD^, GST‐GmCERK1^CD+Km^, GST‐NFR5a^CD^, His‐GmCERK1^CD^, and His‐NFR5a^CD^ proteins. The soluble GST‐fusion proteins were incubated with 50 μL glutathione agarose beads (Invitrogen, Shanghai, China) at 4°C for 2 h. The beads were washed five times and incubated with His‐tagged proteins (1:1, v/v) for another 2 h at 4°C. The beads were washed five times, and the presence of His‐tagged proteins was detected using western blotting and His antibody.

### *In vitro* kinase assay

To detect GmNFR5a^CD^ phosphorylation by GmCERK1^CD^ *in vitro*, 1 mg of purified recombinant His‐GmCERK1^CD^ or His‐GmCERK1^CD+Km^ (negative control) proteins were used as kinases and 3 mg of purified recombinant His‐GmNFR5a^CD^ protein as substrate. Kinases and substrate were incubated in a reaction buffer containing 25 mM Tris‐HCl, pH 7.5, 10 mM MgCl_2_, 1 mM DTT, and 1 mM ATP (Sigma, St. Louis, MI, USA) at 37°C for 2 h. The reaction mixtures were subjected to immunoblotting to detect substrate phosphorylation by using the anti‐pSer/Thr antibody. The same method is used to detect phosphorylation between GmCAK1 and GmCERK1.

### Phytophthora sojae infection assays

For soybean etiolated hypocotyls inoculation assays, etiolated hypocotyls of soybean (cultivar Williams) were inoculated with *P. sojae* zoospore suspensions (100 zoospores). Infected etiolated hypocotyls were maintained at 25°C in the dark for 2 d, photographed and collected for biomass detection. For soybean hairy‐root inoculation assays, transgenic hairy roots were soaked in CTOS/CSOS (50 mg/L) or mock (dH_2_O) for 12 h and then were infected with red fluorescent protein (RFP)‐labeled *P. sojae* strain P6497, and fluorescence was visualized in the infected area of soybean hairy root and the biomass of *P. sojae* was determined by qRT‐PCR at 48 hpi.

### Fluorescence microscopy

Fluorescence was visualized for BiFC, subcellular localization, and soybean hairy‐root transformation analyses using a Zeiss LSM710 scanning confocal microscope with sequential imaging at excitation wavelengths of 488 nm (green/GFP), 514 nm (yellow/yellow fluorescent protein (YFP)), and 633 nm (red/chlorophyll).

### Quantitative RT‐PCR analysis

Total RNA was extracted from 2‐week‐old soybean (cultivar Williams or Zhonghuang13) seedlings using RNA‐easy TM Isolation Reagent (Cat. No. R701‐01; Vazyme). Two micrograms of total RNA were subjected to synthesize cDNA using All‐in‐One 5×RT MasterMix (Cat. No. G592; abm), and quantitative (q)RT‐PCR was performed with BlasTaq 2× qPCR MasterMix (Cat. No. G891; abm) on Bio‐Rad CFX96TM real‐time system. For data analyses, the 2^−ΔΔCt^ method (Livak and Schmittgen, 2001) was used to calculate the value and the mRNA level of GmCYP2 (Glyma.12G024700) (Zhao et al., 2022) was used as an internal reference. The primers used for qRT‐PCR are listed in Table S1.

### Statistical analysis

The data in this study were averages from at least three independent experiments, and the values were subjected to statistical analysis through analysis of variance (ANOVA) followed by Student's *t*‐test or Duncan's honestly significant difference test.

## CONFLICTS OF INTEREST

The authors declare that they have no competing interests.

## AUTHOR CONTRIBUTIONS

Y.C.W., H.Y., Y.W., Y.M.W., S.D., Z.M., K.D., W.Y., and G.S. conceived and conceptualized the study and designed the experiments. Q.Z., J.C., R.Y., X.L., and G.S. performed soybean gene silencing assays, protein interaction assays *in vitro* and *in vivo*, protein phosphorylation assays, and plant immune responses detection assays. Z.Z., K.H., and H.Y. performed all bioinformatics analysis. B.Y., K.D., Q.Z., X.L., X.M., and G.S. performed soybean hairy‐root transformation assays. H.Y., T.L., and W.W. performed chitooligosaccharides purification, protein purification, and MST assays. Y.C.W., H.Y., Y.W., Y.M.W., K.Y., and G.S. wrote the manuscript with input from all authors. All authors have read and approved the contents of this paper.

## Supporting information

Additional Supporting Information may be found online in the supporting information tab for this article: http://onlinelibrary.wiley.com/doi/10.1111/jipb.70042/suppinfo

**Figure S1.** Electrospray ionization mass spectrometry and MALDI‐TOF mass spectrometry were performed to characterize the chitooligosaccharides monomers
**Figure S2.** Analyzing the degree of acetylation of CSOS by NMR spectrum
**Figure S3.** CSOS (mix) triggers soybean ROS production but CSOS (dp4–6) cannot
**Figure S4.** Interrelationships of the orders and some families supported by bootstrap frequencies above 50% in the analyses of angiosperms
**Figure S5.** Phylogenetic analysis of different plant LysMs proteins
**Figure S6.** GmNFR5a and GmCERK1 are essential for chitooligosaccharides‐triggered immune responses and disease resistance in soybean
**Figure S7.** The sequence alignment of GmLYK5 with AtLYK5
**Figure S8.** Expression and purification of proteins
**Figure S9.** The control setup for microscale thermophoresis (MST) detection of the interaction between GmNFR5a/GmCERK1 and CTOS/CSOS
**Figure S10.** Predicted overall structure of GmNFR5a^ECD^ or GmCERK1^ECD^ in complex with CTOS (dp3) or CSOS (dp3)
**Figure S11.** Functional validation of GmCERK1/GmNFR5a binding sites through heterologous expression in *Nicotiana benthamiana* reveals their role in CTOS/CSOS‐induced plant immunity
**Figure S12.** Protein expression analysis of GmCERK1/GmNFR5a mutants in soybean root hairs
**Figure S13.** GmCERK1 and GmNFR5a are plasma membrane‐localized proteins
**Figure S14.** The sequence alignment of GmNFR5a with AtLYK5
**Figure S15.** Evolutionary analysis of CAK1 protein
**Figure S16.** Structure‐based sequence alignment of CAK1 proteins from different species
**Figure S17.** Schematic model for chitooligosaccharides from extracellular binding to intracellular signal transduction
**Table S1.** Primers used in this study

## Data Availability

Sequence data from this article can be found under the following GenBank accession numbers: *GmNFR5a* (Glyma.11G063100), *GmCERK1* (Glyma.20G054500), *GmLYK5* (Glyma.14g077700), *GmCAK1* (Glyma.17G117800), *GmPDS* (Glyma.18G003900). Rice Genome Annotation Project accession numbers: *OsCERK1* (Os09g33630), *OsCEBiP* (Os03g04110), *OsRLCK185* (Os05g0372100), *OsRLCK176* (Os05g0110900). TAIR accession numbers: *AtCERK1* (At3G21630), *AtLYK5* (At2G33580), *AtPBL19* (AT5G47070), *AtPBL27* (AT5G18610), *AtBIK1* (AT2G39660).
