# Supplementary material for: A GmNRF5a–GmCERK1–GmCAK1 module mediates chitin/chitosan‐triggered immune response in soybean
Source: J Integr Plant Biol. 2025 Oct 6;68(1):257–77. doi: 10.1111/jipb.70042 (PMC12782889; doi:10.1111/jipb.70042)
Supplement: Supplementary file 1 — Figure S1. Electrospray ionization mass spectrometry and MALDI‐TOF mass spectrometry were performed to characterize the chitooligosaccharides monomers Figure S2. Analyzing the degree of acetylation of CSOS by NMR spectrum Figure S3. CSOS (mix) triggers soybean ROS production but CSOS (dp4–6) cannot Figure S4. Interrelationships of the orders and some families supported by bootstrap frequencies above 50% in the analyses of angiosperms Figure S5. Phylogenetic analysis of different plant LysMs proteins Figure S6. GmNFR5a and GmCERK1 are essential for chitooligosaccharides‐triggered immune responses and disease resistance in soybean Figure S7. The sequence alignment of GmLYK5 with AtLYK5 Figure S8. Expression and purification of proteins Figure S9. The control setup for microscale thermophoresis (MST) detection of the interaction between GmNFR5a/GmCERK1 and CTOS/CSOS Figure S10. Predicted overall structure of GmNFR5aECD or GmCERK1ECD in complex with CTOS (dp3) or CSOS (dp3) Figure S11. Functional validation of GmCERK1/GmNFR5a binding sites through heterologous expression in Nicotiana benthamiana reveals their role in CTOS/CSOS‐induced plant immunity Figure S12. Protein expression analysis of GmCERK1/GmNFR5a mutants in soybean root hairs Figure S13. GmCERK1 and GmNFR5a are plasma membrane‐localized proteins Figure S14. The sequence alignment of GmNFR5a with AtLYK5 Figure S15. Evolutionary analysis of CAK1 protein Figure S16. Structure‐based sequence alignment of CAK1 proteins from different species Figure S17. Schematic model for chitooligosaccharides from extracellular binding to intracellular signal transduction Table S1. Primers used in this study [file JIPB-68-257-s001.doc]

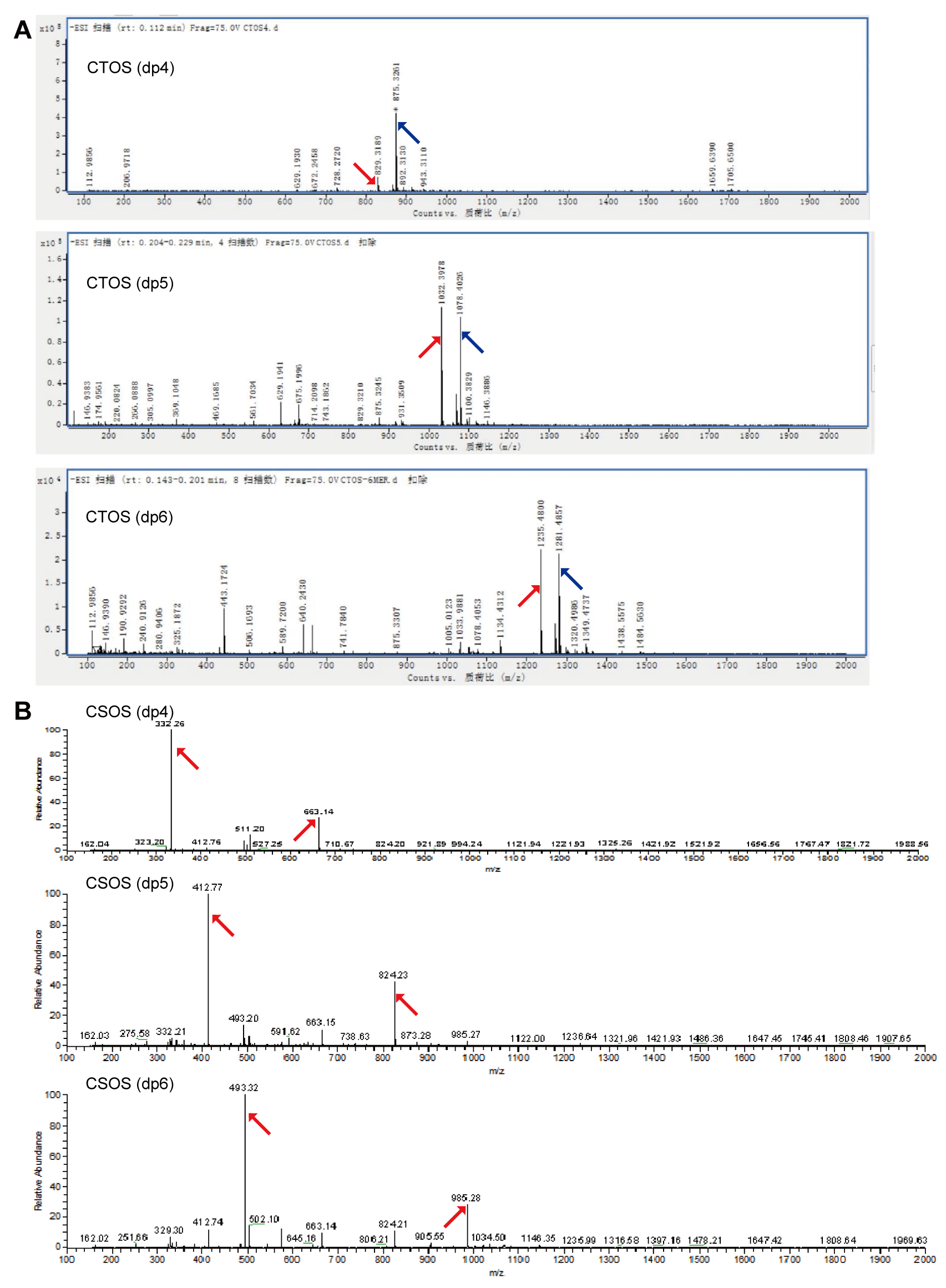


**Figure S1. Electrospray ionization mass spectrometry and MALDI-TOF Mass Spectrometry were performed to characterize the chitooligosaccharides monomers.**

**(A)** The MALDI-TOF Mass Spectrometry of the prepared CTOS monomers (dp4, dp5, and dp6). The arrows represent the monomers of the corresponding CTOS. The red arrows indicate the negative ion peaks of CTOS, and the blue arrows indicate the adduct ion peaks formed by the participation of formic acid.

**(B)** The Electrospray ionization mass spectrometry analysis of the prepared CSOS monomers (dp4, dp5, and dp6). The red arrows indicate the corresponding CSOS monomers. The appearance of doublet peaks results from the simultaneous detection of singly- and doubly-charged ions of the monomer, where the m/z value of the doubly-charged peak is half that of the singly-charged peak.


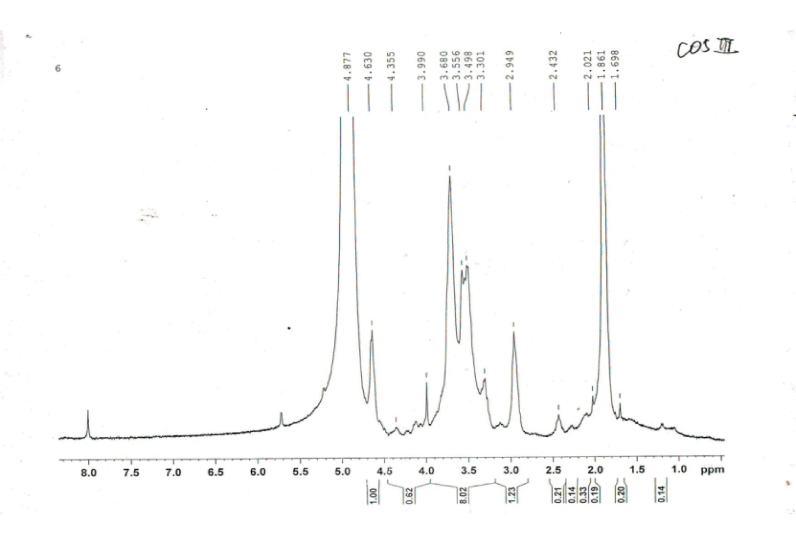


**Figure S2. Analyzing the degree of acetylation of CSOS by nuclear magnetic resonance (NMR) spectrum.**

According to the NMR spectrum, the value at *ca.* δ 2.02 ppm is assigned to H of -CH3 of the N-acetyl group in GlcNAc. The shifts of the protons attached to positions C2-C6 on the sugar ring appear at *ca.* 2.65-4.40 ppm. The deacetylation degree of CSOS sample was determined according to below equation: DD(%) = [1 - (A2/3)/(A1/6)] × 100 = [1 - (0.19/3)/ (1.23 + 8.02 + 0.62)/6] × 100 = 96.14%. A1 are the protons integral values of positions C2-C6 on the sugar ring and A2 are the protons integral values of the three N-acetyl protons of GlcNAc. The above data showed deacetylation degree of CSOS sample is higher than 95%, thus eliminating the uncertain impact of acetylated groups.


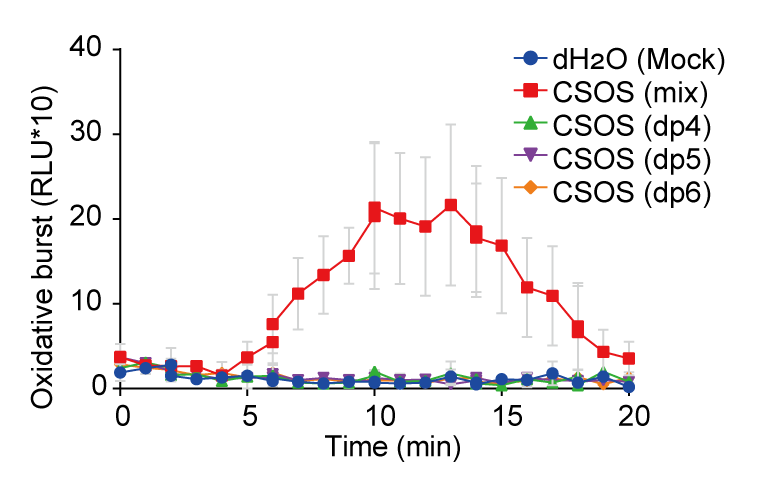


**Figure S3. CSOS (mix) triggers soybean ROS production but CSOS (dp4-6) can not.**


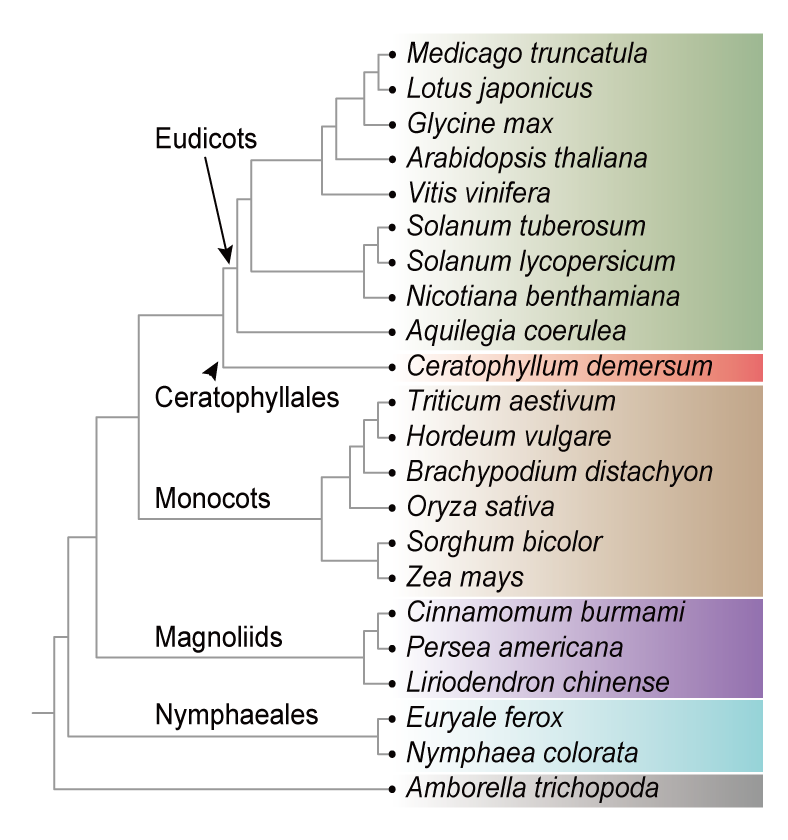


**Figure S4. Interrelationships of the orders and some families supported by bootstrap frequencies above 50% in the analyses of angiosperms.**


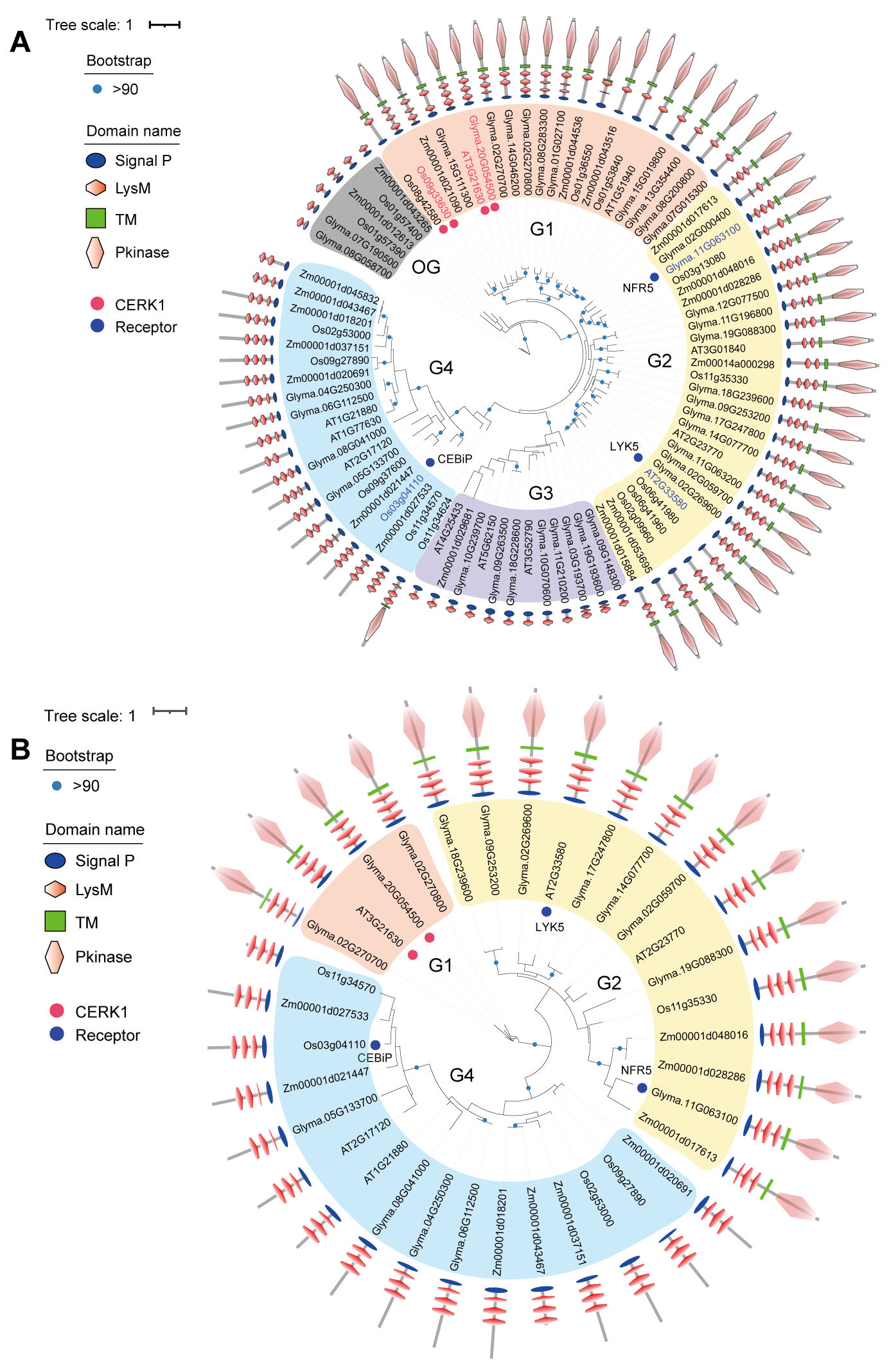


**Figure S5. Phylogenetic analysis of different plant LysMs proteins.**

**(A)** Phylogenetics tree of 84 LysMs family membrane proteins from different plants, including soybean, Arabidopsis, rice, and maize. Bootstrap values (%) obtained from 1000 trials are indicated at nodes.

**(B)** Phylogenetics tree of 34 LysMs family membrane proteins based on LysM2 domain sequence alignment.


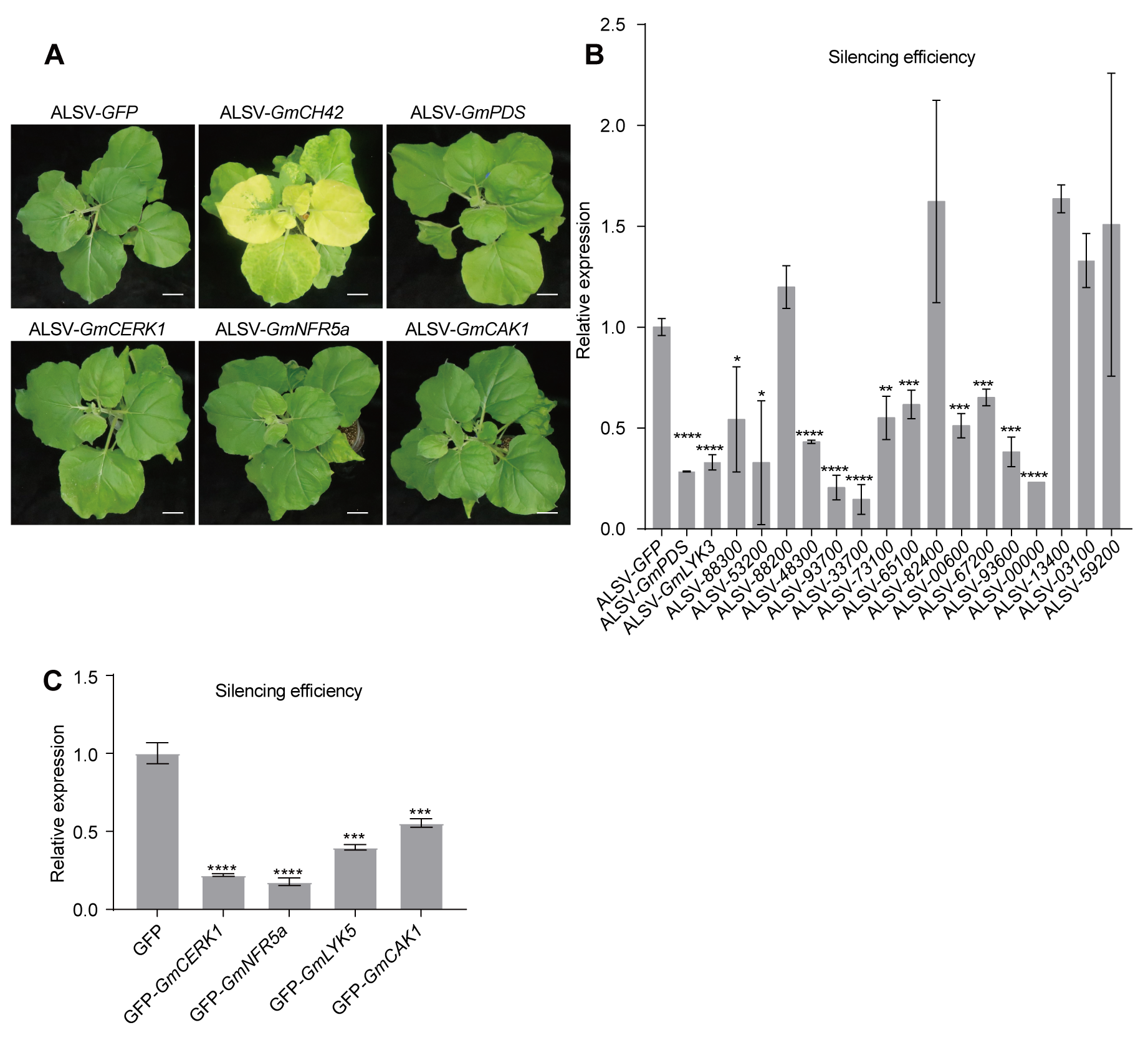


**Figure S6. GmNFR5a and GmCERK1 are essential for chitooligosaccharides-triggered immune responses and disease resistance in soybean.**

**(A)** Plant growth phenotype expressed recombinant ALSV-*GFP*, ALSV-*GmCH42*, ALSV-*GmPDS*, ALSV-*GmCERK1*, ALSV-*GmNFR5a*, and ALSV-*GmCAK1* in *N. benthamiana*. *N. benthamiana* leaves expressing ALSV-*GmCH42* is yellow. After enriching virus particles on *N. benthamiana*, the soybean leaves were inoculated with virus particles through friction.

**(B)** Silencing efficiency of partial *GmLysMs* gene is quantified by real-time quantitative polymerase chain reaction (qRT-PCR) measurement, normalized with *GmCYP2*, and expressed as mean fold changes relative to ALSV-*GFP* treated leaves, which is set as 1.

**(C)** Silencing efficiency of *GmLysMs* and *GmCAK1* genes in soybean hairy roots is quantified by real-time quantitative polymerase chain reaction (qRT-PCR) measurement, normalized with *GmCYP2*, and expressed as mean fold changes relative to ALSV-*GFP* treated leaves, which is set as 1.


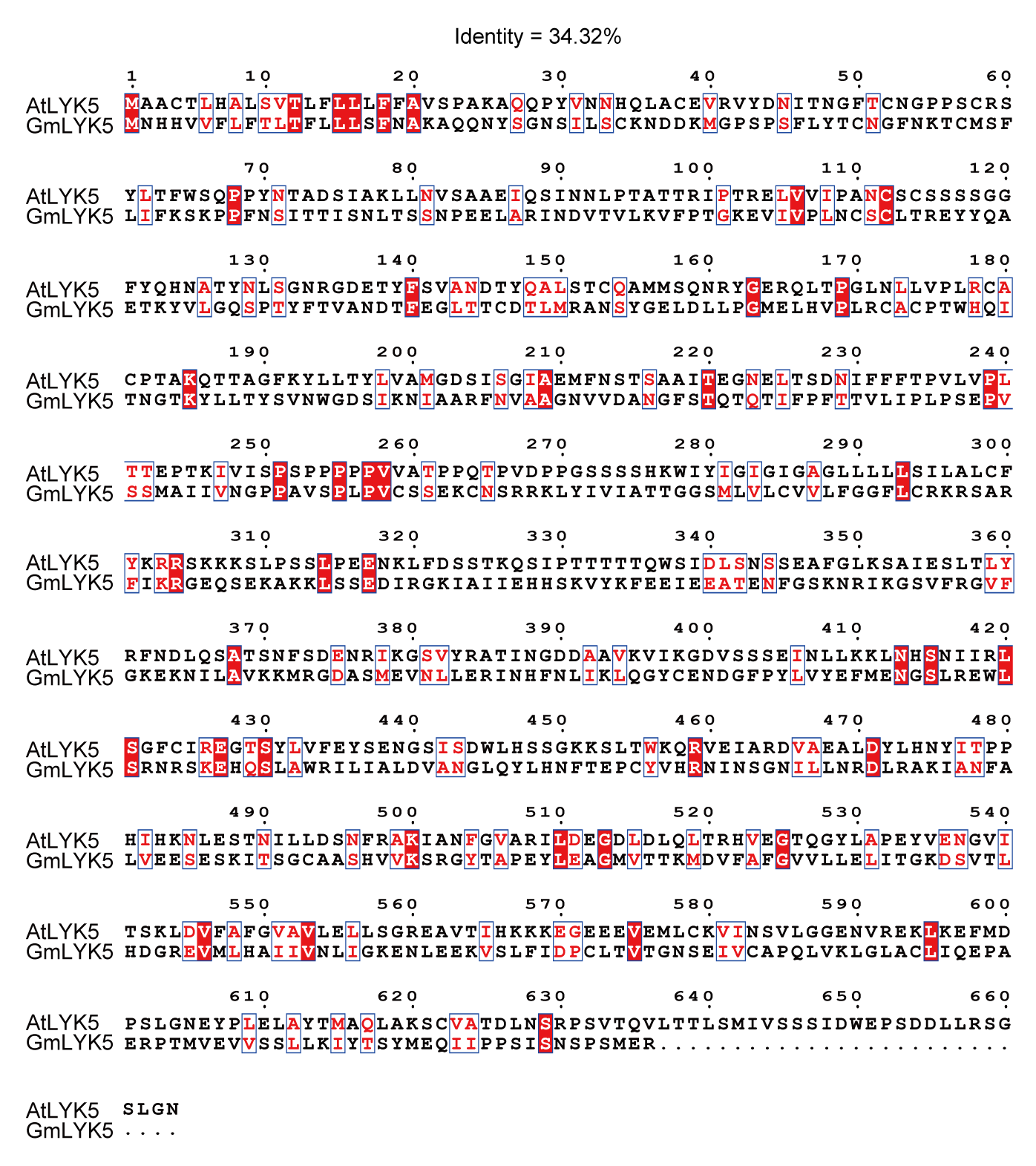


**Figure S7. The sequence alignment of GmLYK5 with AtLYK5**


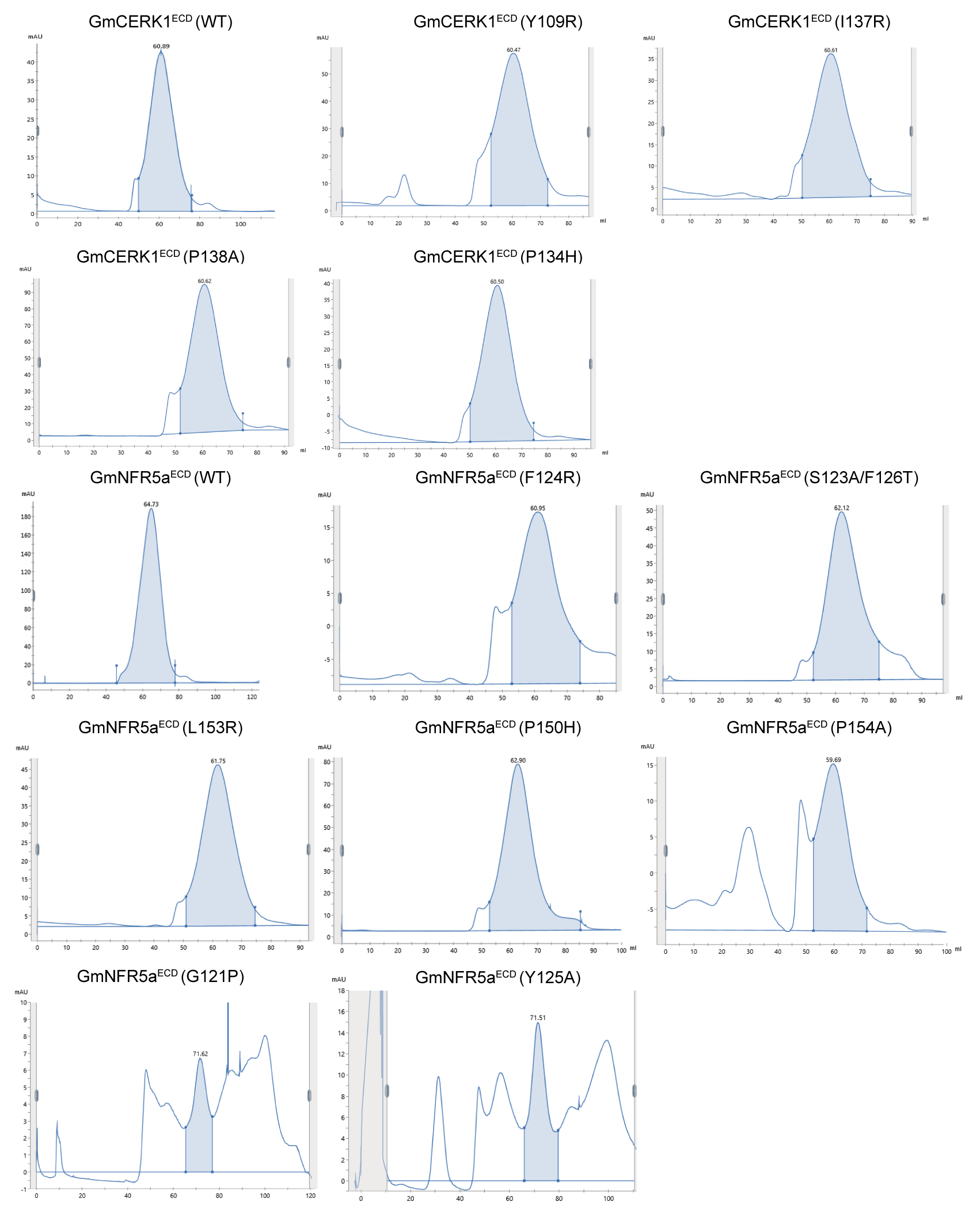


**Figure S8. Expression and purification of proteins.**

GmCERK1^ECD^, GmNFR5a^ECD^, and their mutants were expressed in *Pichia pastoris* strain X-33 and purified by a HiTrap Q FF column and size exclusion chromatography with a HiLoad Superdex 200 pg column.


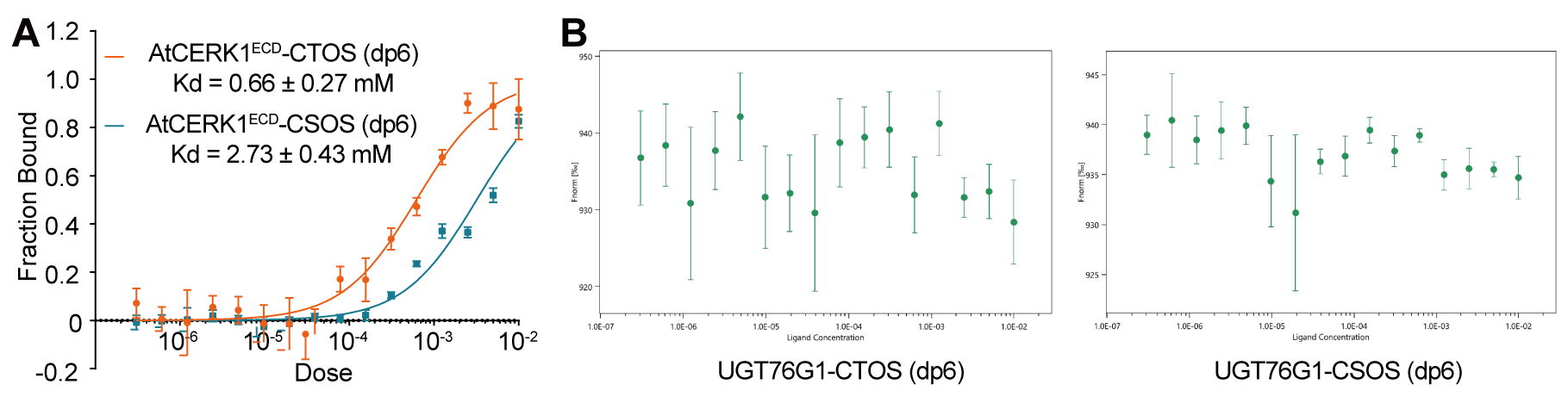


**Figure S9. The control setup for MST detection of the interaction between GmNFR5a/GmCERK1 and CTOS/CSOS.**

AtCERK1^ECD^ binds to CTOS (dp6) or CSOS (dp6) *in vitro* by MST as a positive control **(A)**. UGT76G1 as a negative control cannot bind to CTOS (dp6) (left) or CSOS (dp6) (right) by MST **(B)**.


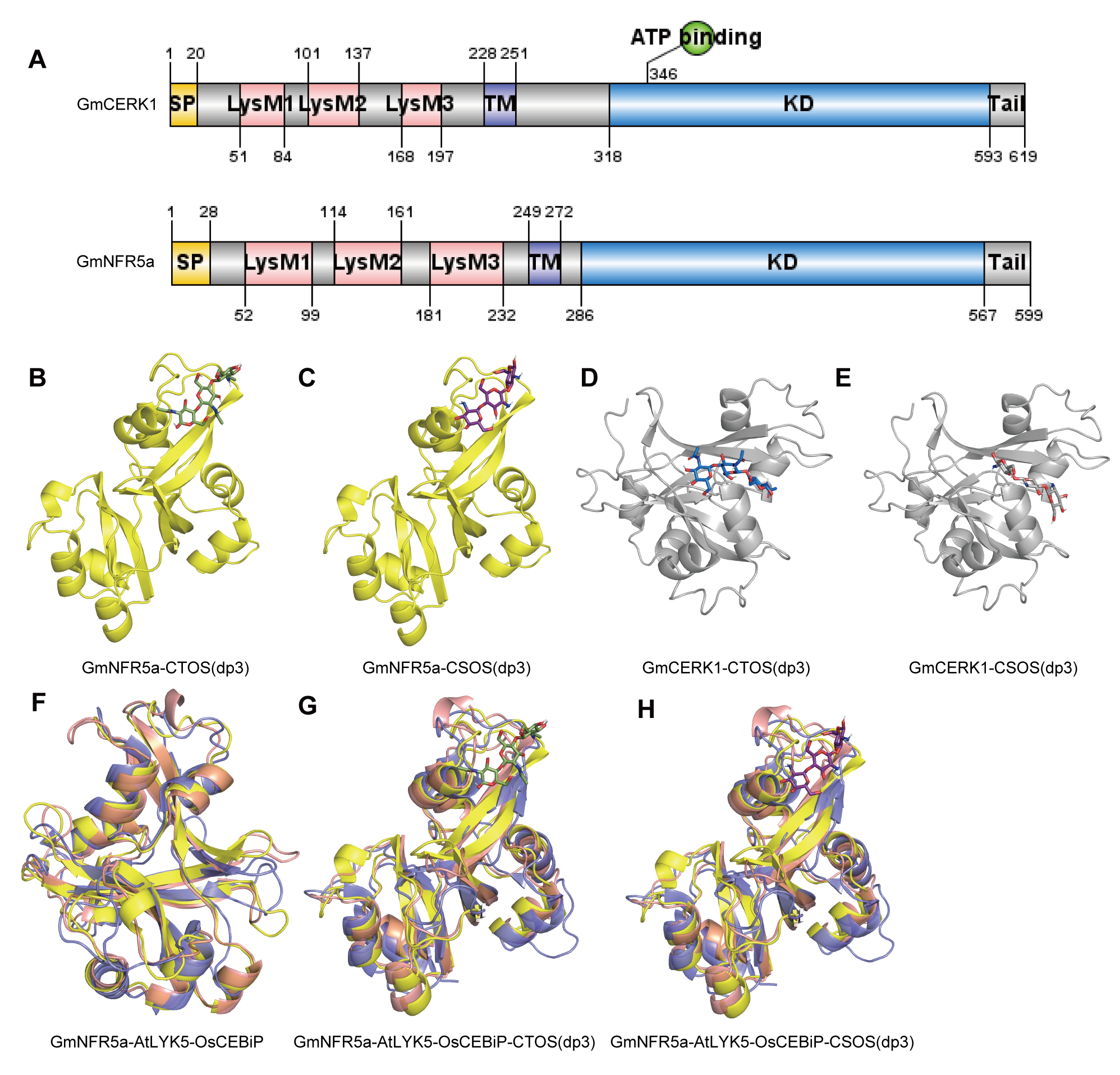


**Figure S10. Predicted overall structure of GmNFR5a^ECD^ or GmCERK1^ECD^ in complex with CTOS (dp3) or CSOS (dp3).**

**(A)** Schematic representation of the domain architecture of GmCERK1 and GmNFR5a. GmCERK1 (top) contains a signal peptide (SP), three LysM domains (LysM1-3), a transmembrane domain (TM), a kinase domain (KD) containing an ATP binding site, and a C-terminal domain (Tail), which are shown as different colors. GmNFR5a (bottom) contains a signal peptide (SP), three LysM domains (LysM1-3), a transmembrane domain (TM), a kinase domain (KD), and a C-terminal domain (Tail).

**(B-C)** The overall structure of GmNFR5a^ECD^ in complex with CTOS (dp3) **(B)** or CSOS (dp3) **(C)**. The GmNFR5a^ECD^ structure are shown in yellow.

**(D-E)** The overall structure of GmCERK1^ECD^ in complex with CTOS (dp3) (**D**) or CSOS (dp3) (**E**). The GmCERK1^ECD^ structure are shown in silvery.

**(F)** The structural superposition of GmNFR5a^ECD^, AtLYK5^ECD^, and OsCEBiP^ECD^.

GmNFR5a^ECD^, AtLYK5^ECD^, and OsCEBiP^ECD^ structures are shown in yellow, purple, and pink, respectively.

**(G-H)** The similar chitooligosaccharides binding areas shared with different chitin-recognition receptors, showing structural superposition of CTOS (dp3)- **(G)** or CSOS (dp3)-bound **(H)** GmNFR5a, AtLYK5, and OsCEBiP.


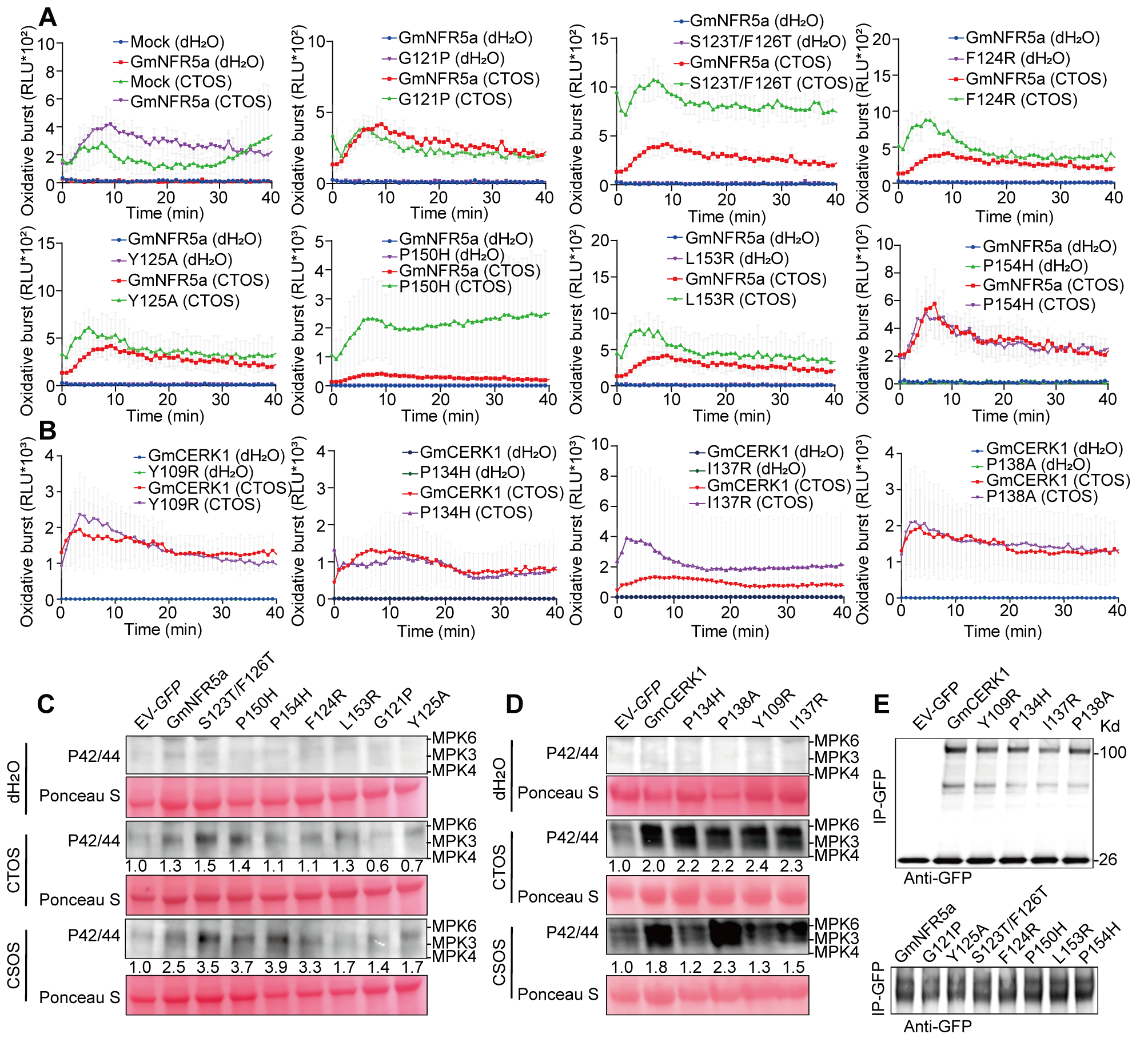


**Figure S11. Functional validation of GmCERK1/GmNFR5a binding sites through heterologous expression in *Nicotiana benthamiana* reveals their role in CTOS/CSOS-induced plant immunity.**

**(A)** ROS production triggered by CTOS (50 mg L^-1^) in *N. benthamiana* leaves overexpressing *GmNFR5a*, *G121P*, *S123T/F126T*, *F124R*, *Y125A*, *P150H*, *L153R*, and *P154H*. Mean RLU (Relative Luminescence Unit) (± *SD*) are shown (*n* = 6).

**(B)** ROS production triggered by CTOS (50 mg L^-1^) in *N. benthamiana* leaves overexpressing *GmCERK1*, *Y109R*, *P134H*, *I137R*, and *P138A*. Mean RLU (Relative Luminescence Unit) (± *SD*) are shown (*n* = 6).

**(C-D)** CTOS/CSOS triggered MAPK phosphorylation in overexpressed-*GmNFR5a*, *G121P*, *S123T/F126T*, *F124R*, *Y125A*, *P150H*, *L153R*, and *P154H* **(C)** and overexpressed-*GmCERK1*, *Y109R*, *P134H*, *I137R*, and *P138A* **(D)** *N. benthamiana* at 10 min. Total protein was analysed by immunoblot with an antibody for phosphorylated MPK6/3/4 (P42/44). The total band intensities were quantified using Image J.

**(E)** Western blot analysis of *GmCERK1*/*GmNFR5a* expression levels.


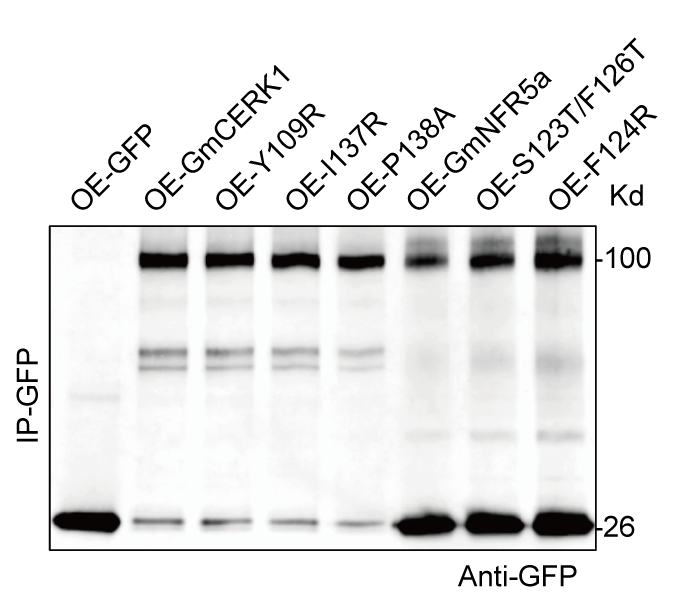


**Figure S12. Protein expression analysis of GmCERK1/GmNFR5a mutants in soybean root hairs.**


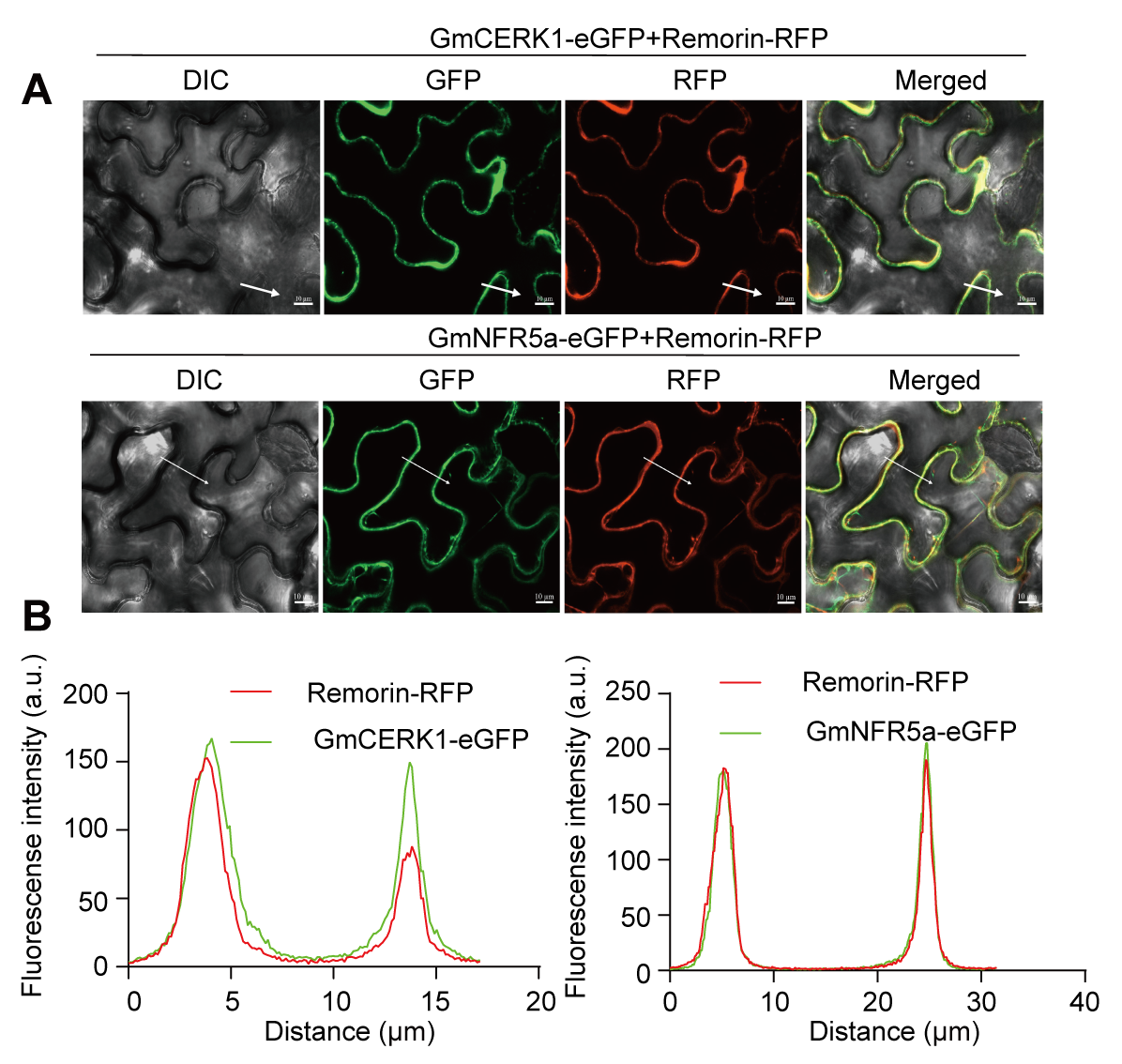


**Figure S13. GmCERK1 and GmNFR5a are plasma membrane-localized proteins.**

**(A)** GmCERK1 and GmNFR5a localize to the plasma membrane. Expression of GmCERK1-eGFP or GmNFR5a-eGFP fusion proteins in *N. benthamiana* through agro-infiltration revealed that GmCERK1 or GmNFR5a is localized at the cell periphery. The Remorin protein as a membrane localization marker was used as a positive control. Fluorescence from epidermal cells in the infiltrated tissues was observed by confocal microscopy at 24 hpi. Scale bars, 10 mm.

**(B)** Fluorescence intensity profiles of GmCERK1-eGFP (left) or GmNFR5a-eGFP (right) with Remorin-RFP in membrane transects (white arrowheads). y axis, GFP or RFP relative fluorescence intensity; x axis, transect length (mm).


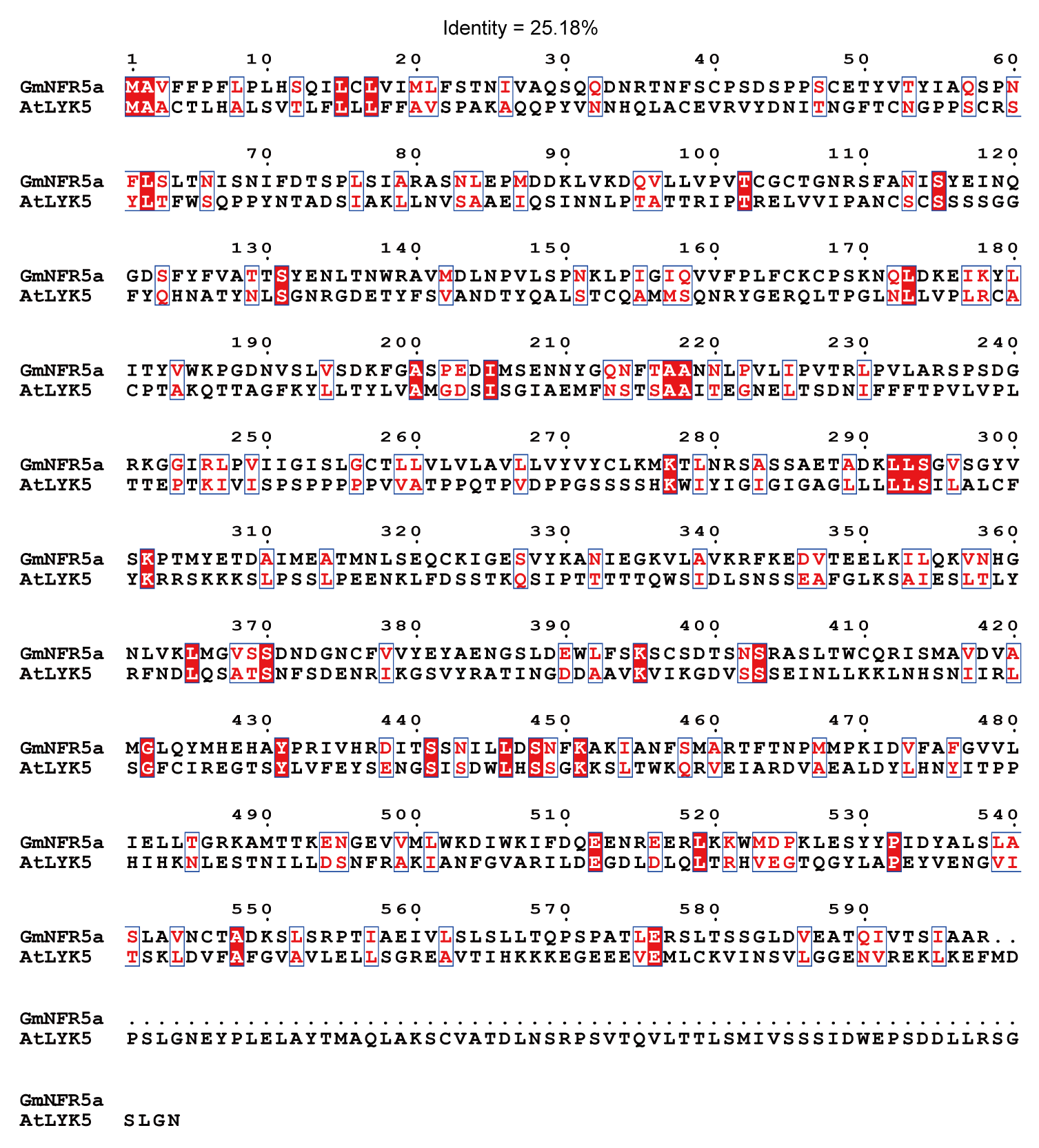


**Figure S14. The sequence alignment of GmNFR5a with AtLYK5.**


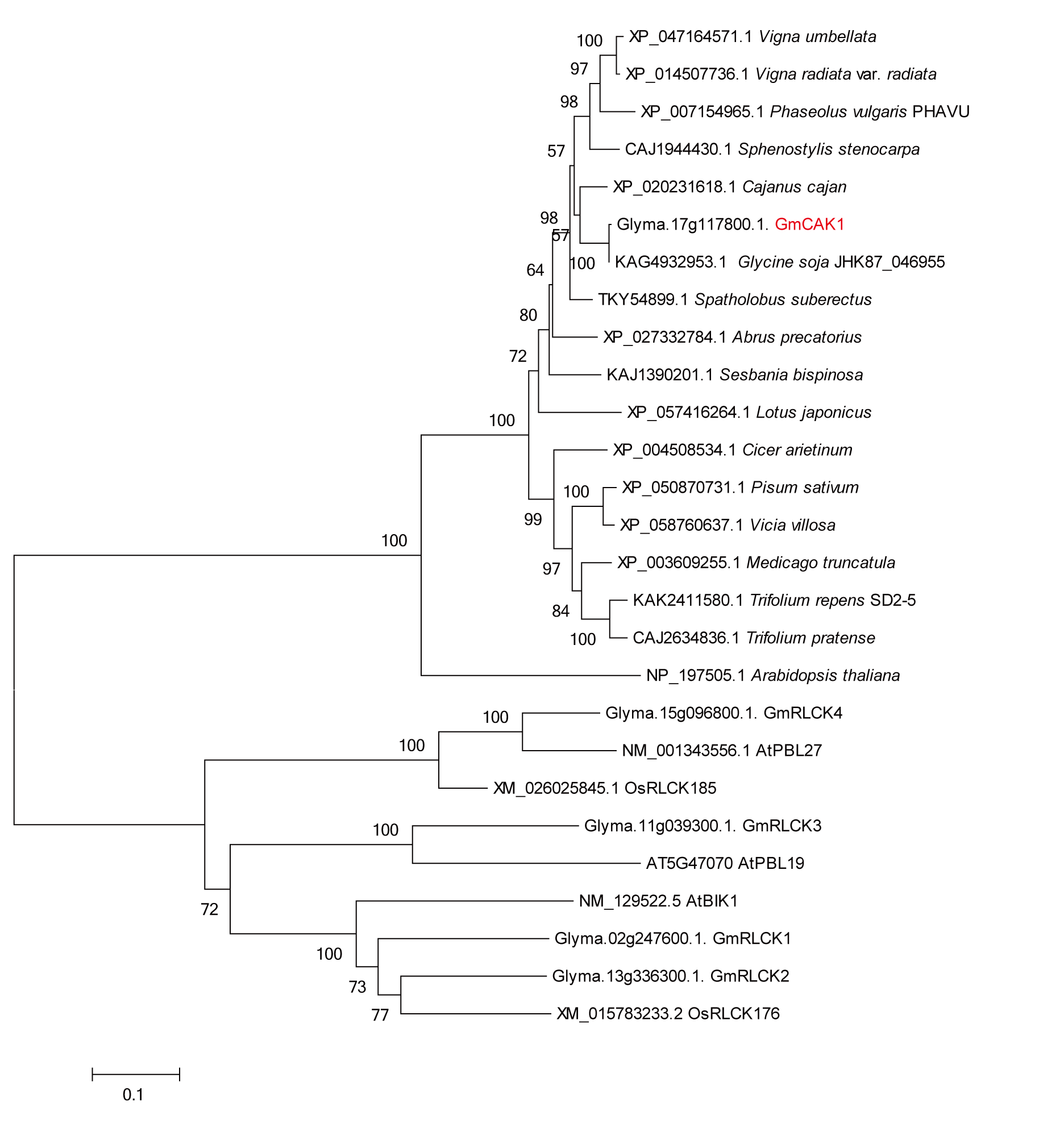


**Figure S15. Evolutionary analysis of CAK1 protein.**

Bootstrap values (%) obtained from 1000 trials are indicated at nodes.


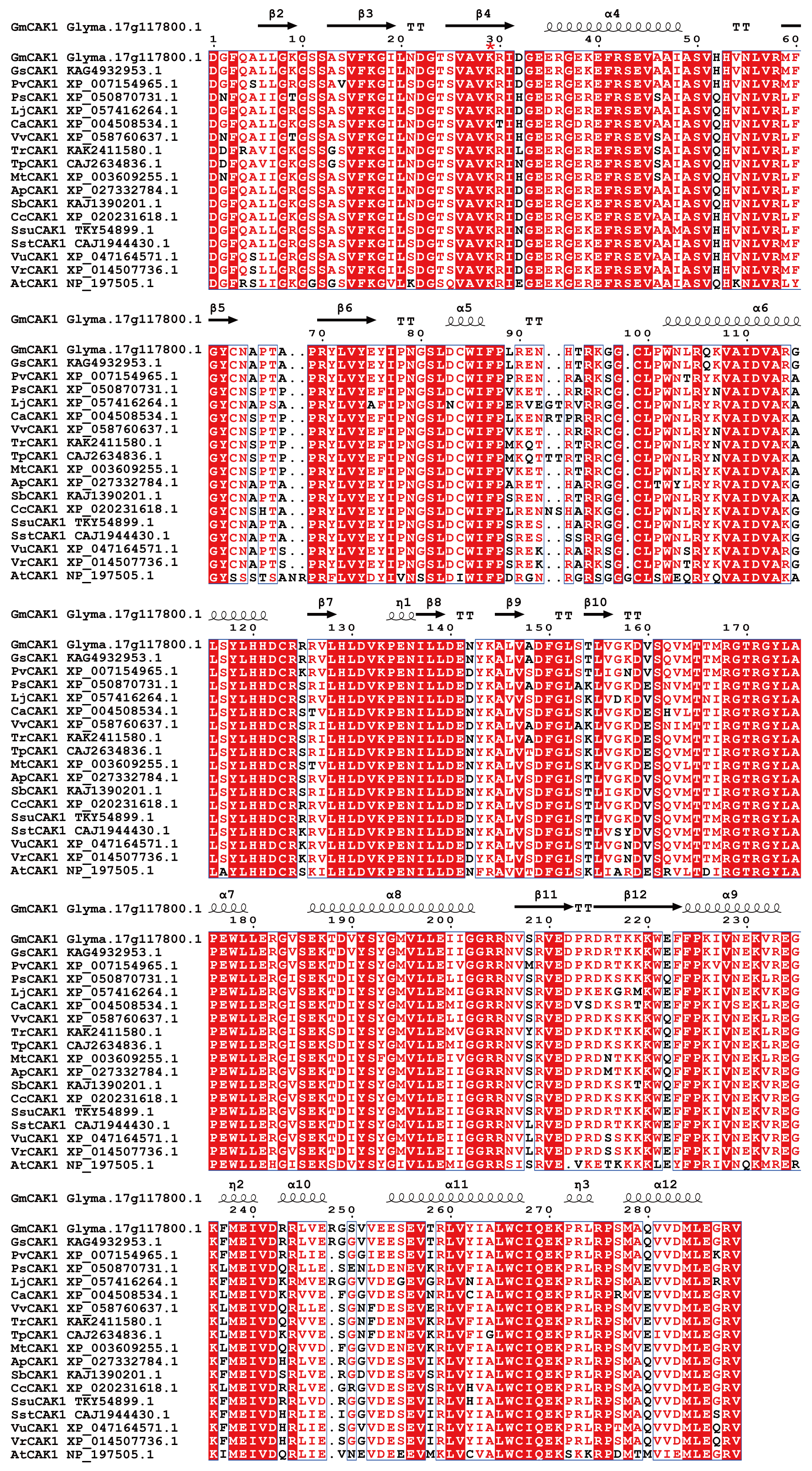


**Figure S16. Structure based sequence alignment of CAK1 proteins from different species.**

Secondary structure elements in GmCAK1 are marked on the top.


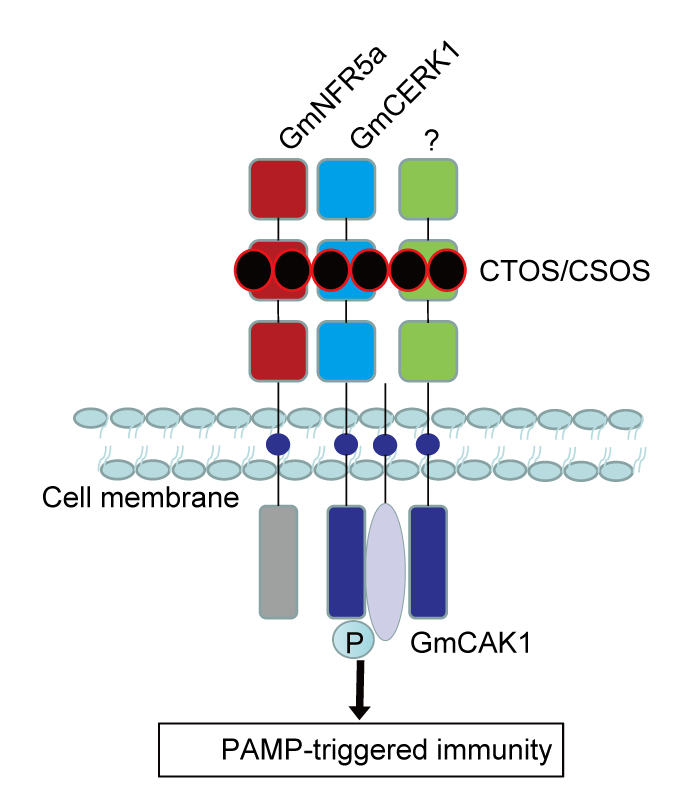


**Figure S17. Schematic model for chitooligosaccharides from extracellular binding to intracellular signal transduction.**

CTOS (dp6) or CSOS (dp6) binds to GmNFR5a and GmCERK1 complex. The binding ability of CTOS to GmNFR5a is weaker than that of GmCERK1, while the binding ability of CSOS to GmNFR5a is comparable to that of GmCERK1. We speculate that there are other proteins on the soybean cell membrane responsible for recognizing CTOS and CSOS. GmNFR5a and GmCERK1 form a heterotetramer. Activated GmCERK1 interacts with and phosphorylates GmCAK1, which is a direct downstream target of GmCERK1.

**Table S1. Primers used in this study**

**Primers used for Real-time RT-PCR**

| **Primer** | **Sequences** | **Usage** |
| --- | --- | --- |
| GmCYP2-qFP | CGGGACCAGTGTGCTTCTTCA | Real-time RT-PCR of GmCYP2 |
| GmCYP2-qRP | CCCCTCCACTACAAAGGCTCG |
| PsActin-qFP | ACTGCACCTTCCAGACCATC | Real-time RT-PCR of PsActin |
| PsActin-qRP | CCACCACCTTGATCTTCATG |
| GmPDS-qFP | CCTGAACGGGTAACTGATGAGGTG | Real-time RT-PCR of GmPDS in VIGS |
| GmPDS-qRP | CGGCATACAAAGTCTTTCGGGTG |
| GmNFR5a-qFP | GGTGACAATGTTTCCCTTG | Real-time RT-PCR of GmNFR5a in VIGS |
| GmNFR5a-qRP | AGAACCAGTAGCGTGCATC |
| GmCERK1-qFP | TCGGCTAAGGAAGCAAT | Real-time RT-PCR of GmCERK1 in VIGS |
| GmCERK1-qRP | GTGCCATCTTGCGAACTG |
| GmCAK1-qFP | GGAAATTCATGGAGATTGT | Real-time RT-PCR of GmCAK1 in VIGS |
| GmCAK1-qRP | ATCAACAGCAAGCAAATCA |
| GmLYK5-qFP | CAGTGAGATAGTATGTGCTC | Real-time RT-PCR of GmLYK5 in VIGS |
| GmLYK5-qRP | CCATGCTAGGACTGTTGCT |
| GmNFR5a-qFP | TACATACGTGTGGAAGCCCG | Real-time RT-PCR of GmNFR5a in hairy-root transformation |
| GmNFR5a-qRP | CTTCCGTCCGAAGGAGATCG |
| GmCERK1-qFP | CGAACGATTCTGTTGCCGAC | Real-time RT-PCR of GmCERK1 in hairy-root transformation |
| GmCERK1-qRP | TTGGACGGAGTTGGGGATTG |
| GmLYK5-qFP | GATCCTTGCCTCACTGTCACT | Real-time RT-PCR of GmLYK5 in hairy-root transformation |
| GmLYK5-qRP | GGTTGGTCTCTCTGCTGGTT |
| GmCAK1-qFP | GGTCTCTCAACGCTTGTTG | Real-time RT-PCR of GmCAK1 in hairy-root transformation |
| GmCAK1-qRP | CAACAGAACCATCCCATAGC |

**Primers used for constructs in soybean hairy-root transformation, virus inducing gene silence**

| **Primer** | **Sequences** | **Usage** |
| --- | --- | --- |
| pFGC5941-GFP-GmNFR5a-1FP | TTACAATTACCATGGGGCGCGCCATGGCTGTCTTCTTTCCC | Vector construct of pFGC5941-GFP-GmNFR5a |
| pFGC5941-GFP-GmNFR5a-1RP | TTAAATCATCGATTGGGCGCGCCTGGTACGAGTAAGACTTGGT |
| pFGC5941-GFP-GmNFR5a-2FP | CTCTAGACTCACCTAGGATCCATGGCTGTCTTCTTTCCC |
| pFGC5941-GFP-GmNFR5a-2RP | AATTTGCAGGTATTTGGATCCTGGTACGAGTAAGACTTGGT |
| pFGC5941-GFP-GmCERK1-1FP | TTACAATTACCATGGGGCGCGCCACGCTGAATGTTACGGTT | Vector construct of pFGC5941-GFP-GmCERK1 |
| pFGC5941-GFP-GmCERK1-1RP | TTAAATCATCGATTGGGCGCGCCCAGAAGAGCTGCCACAAT |
| pFGC5941-GFP-GmCERK1-2FP | CTCTAGACTCACCTAGGATCCACGCTGAATGTTACGGTT |
| pFGC5941-GFP-GmCERK1-2RP | AATTTGCAGGTATTTGGATCCCAGAAGAGCTGCCACAAT |
| pFGC5941-GFP-GmLYK5-1FP | TTACAATTACCATGGGGCGCGCCGCCATAATTGTCAATGGTCCA | Vector construct of pFGC5941-GFP-GmLYK5 |
| pFGC5941-GFP-GmLYK5-1RP | TTAAATCATCGATTGGGCGCGCCTATCTTGCCGCGGATATC |
| pFGC5941-GFP-GmLYK5-2FP | CTCTAGACTCACCTAGGATCCGCCATAATTGTCAATGGTCCA |
| pFGC5941-GFP-GmLYK5-2RP | AATTTGCAGGTATTTGGATCCTATCTTGCCGCGGATATC |
| pFGC5941-GFP-GmCAK1-1FP | TTACAATTACCATGGGGCGCGCCGTGGAGCGTGGGAGTGTTG | Vector construct of pFGC5941-GFP-GmCAK1 |
| pFGC5941-GFP-GmCAK1-1RP | TTAAATCATCGATTGGGCGCGCCACCAGATAAAATAGTGTTTG |
| pFGC5941-GFP-GmCAK1-2FP | CTCTAGACTCACCTAGGATCCGTGGAGCGTGGGAGTGTTG |
| pFGC5941-GFP-GmCAK1-2RP | AATTTGCAGGTATTTGGATCCACCAGATAAAATAGTGTTTG |
| ALSV-GmNFR5a-FP | GATTTCACACTCGAGCCCGGGATGGCTGTCTTCTTTCCCTTTCT | Vector construct of ALSV-GmNFR5a |
| ALSV-GmNFR5a-RP | TTCTAGCAGGGATCCCCCGGGTGGACTTAGAACGGGGTTTAAATC |
| ALSV-GmCERK1-FP | GATTTCACACTCGAGCCCGGGATGGAAGCCTTGAGGTTGGC | Vector construct of ALSV-GmCERK1 |
| ALSV-GmCERK1-RP | TTCTAGCAGGGATCCCCCGGGAGAGCAATTAACCGTAACATTCAGC |
| ALSV-GmLYK5-FP | GATTTCACACTCGAGCCCGGGAACATATTGGCTGTCAAGAAAATGA | Vector construct of ALSV-GmLYK5 |
| ALSV-GmLYK5-RP | TTCTAGCAGGGATCCCCCGGGCATCCCTGCCTCCAGATACTCC |
| ALSV-GmCAK1-FP | GATTTCACACTCGAGCCCGGGGATTGTAGGAGGAGGGTTTTGCA | Vector construct of ALSV-GmCAK1 |
| ALSV-GmCAK1-RP | TTCTAGCAGGGATCCCCCGGGCTTCTCTTGTATGCACCACAATGC |

**Primers used for constructs in Co-IP, *in vitro* pull-down, *in vitro* kinase assays**

| **Primer** | **Sequences** | **Usage** |
| --- | --- | --- |
| pBIN-GFP-GmCERK1-FP | TTACGAACGATAGCCGGTACCATGGAAGCCTTGAGGTTG | Vector construct of pBIN-GFP-GmCERK1 |
| pBIN-GFP-GmCERK1-RP | GCCCTTGCTCACCATCCCGGGTCTTCCGGACATAAGATTCACAAG |
| pBIN-GFP-GmNFR5a-FP | TTACGAACGATAGCCGGTACCATGGCTGTCTTCTTTCCCTTTC | Vector construct of pBIN-GFP-GmNFR5a |
| pBIN-GFP-GmNFR5a-RP | GCCCTTGCTCACCATCCCGGGACGAGCTGCTATGGAAGTGA |
| pBIN-GFP-RXEG1-FP | TTACGAACGATAGCCGGTACCATGGGCAAAAGGGAATATCCA | Vector construct of pBIN-GFP-RXEG1 |
| pBIN-GFP-RXEG1-RP | GCCCTTGCTCACCATCCCGGGAGCCCTTAACTTTCTCTTCAGT |
| pBIN-HA-GmCERK1-FP | TTACGAACGATAGCCGGTACCATGGAAGCCTTGAGGTTG | Vector construct of pBIN-HA-GmCERK1 |
| pBIN-HA-GmCERK1-RP | AACATCGTATGGGTACCCGGGTCTTCCGGACATAAGATTCACAAG |
| pBIN-HA-GmNFR5a-FP | ACGATAGCCGGTACCCCCGGGATGGCTGTCTTCTTTCCCTTTCT | Vector construct of pBIN-HA-GmNFR5a |
| pBIN-HA-GmNFR5a-RP | AACATCGTATGGGTACCCGGGACGAGCTGCTATGGAAGTGACA |
| pBIN-HA-GmCAK1-FP | TTACGAACGATAGCCGGTACCATGGAGGACAGAAAAGCCAACA | Vector construct of pBIN-HA-GmCAK1 |
| pBIN-HA-GmCAK1-RP | AACATCGTATGGGTACCCGGGTCTACCAGATAAAATAGTGTTTG |
| pBIN-HA-BAK1-FP | TTACGAACGATAGCCGGTACCATGGATCAATGGATATTGGGGA | Vector construct of pBIN-HA-BAK1 |
| pBIN-HA-BAK1-RP | AACATCGTATGGGTACCCGGGTCTTGGCCCTGATAACTCATC |
| pGEX-4T-2-GST-GmCERK1^CD^-FP | GGATCCCCAGGAATTCCCGGGCGTAAGAAGATACAGAAGG | Vector construct of pGEX-4T-2-GST-GmCERK1^CD^ |
| pGEX-4T-2-GST-GmCERK1^CD^-RP | GGCCGCTCGAGTCGACCCGGGTCTTCCGGACATAAGATTCAC |
| pGEX-4T-2-GST-GmCERK1^CD+Km^-1FP | GGATCCCCAGGAATTCCCGGGCGTAAGAAGATACAGAAGG | Vector construct of pGEX-4T-2-GST-GmCERK1^CD+Km^ |
| pGEX-4T-2-GST-GmCERK1^CD+Km^-1RP | TGATGCTTGCATATCCATTTCTTCGATTGCGGCTTTCTCTCCCCT |
| pGEX-4T-2-GST-GmCERK1^CD+Km^-2FP | GAAGAAATGGATATGCAAGCATCA |
| pGEX-4T-2-GST-GmCERK1^CD+Km^-2RP | GGCCGCTCGAGTCGACCCGGGTCTTCCGGACATAAGATTCAC |
| pGEX-4T-2-GST-GmNFR5a^CD^-FP | GGATCCCCAGGAATTCCCGGGTATTGTCTGAAAATGAAGACTTTG | Vector construct of pGEX-4T-2-GST-GmNFR5a^CD^ |
| pGEX-4T-2-GST-GmNFR5a^CD^-RP | GGCCGCTCGAGTCGACCCGGGACGAGCTGCTATGGAAGTGACAA |
| pET28-His-GmCERK1^CD^-FP | CAGCAAATGGGTCGCGGATCCCGTAAGAAGATACAGAAGGATGA | Vector construct of pET28-His-GmCERK1^CD^ |
| pET28-His-GmCERK1^CD^-RP | ACGGAGCTCGAATTCGGATCCTCTTCCGGACATAAGATTCACAAGA |
| pET28-His-GmCERK1^CD+Km^-1FP | CAGCAAATGGGTCGCGGATCCCGTAAGAAGATACAGAAGGATGA | Vector construct of pET28-His-GmCERK1^CD+Km^ |
| pET28-His-GmCERK1^CD+Km^-1RP | TGATGCTTGCATATCCATTTCTTCGATTGCGGCTTTCTCTCCCCT |
| pET28-His-GmCERK1^CD+Km^-2FP | GAAGAAATGGATATGCAAGCATCA |
| pET28-His-GmCERK1^CD+Km^-2RP | ACGGAGCTCGAATTCGGATCCTCTTCCGGACATAAGATTCACAAGA |
| pET32-His-GmNFR5a^CD^-FP | GACAAGGCCATGGCTGATATCTATTGTCTGAAAATGAAGACTTTG | Vector construct of pET32-His-GmNFR5a^CD^ |
| pET32-His-GmNFR5a^CD^-RP | CTCGAATTCGGATCCGATATCACGAGCTGCTATGGAAGTGACAA |
| pET32-His-GmCERK1^CD^-FP | GACAAGGCCATGGCTGATATCCGTAAGAAGATACAGAAGG | Vector construct of pET32-His-GmCERK1^CD^ |
| pET32-His-GmCERK1^CD^-RP | CTCGAATTCGGATCCGATATCTCTTCCGGACATAAGATTCAC |
| pET32-His-GmCAK1^CD^-FP | GACAAGGCCATGGCTGATATCAGGCACCGTTACAACCACAGAAG | Vector construct of pET32-His-GmCAK1^CD^ |
| pET32-His-GmCAK1^CD^-RP | CTCGAATTCGGATCCGATATCTCTACCAGATAAAATAGTGT |
| pET32-His-GmCAK1^CD+Km^-1FP | GACAAGGCCATGGCTGATATCAGGCACCGTTACAACCACAGAAG | Vector construct of pET32-His-GmCAK1^CD+Km^ |
| pET32-His-GmCAK1^CD+Km^-1RP | CTCGCTCCTCGCCGTCGATTCGTTCCACCGCAACCGAAGTTCCA |
| pET32-His-GmCAK1^CD+Km^-2FP | GAACGAATCGACGGCGAGGAGCGAGG |
| pET32-His-GmCAK1^CD+Km^-2RP | CTCGAATTCGGATCCGATATCTCTACCAGATAAAATAGTGT |
